# Sarcopenic Obesity and Longitudinal Trajectories on Cognitive Performance and Subtle Cognitive Impairment Over 6 Years in Older Adults

**DOI:** 10.1002/jcsm.70158

**Published:** 2025-12-08

**Authors:** Héctor Vázquez‐Lorente, Indira Paz‐Graniel, Hernando J. Margara‐Escudero, Miguel Ángel Martínez‐González, Dora Romaguera, D. Martinez Urbistondo, Ramon Estruch, Vicente Martín Sánchez, Josep Vidal, Montserrat Fitó, Nuria Goñi, Alice Chaplin, M. Angeles Zulet, Emilio Sacanella, José Antonio de Paz Fernández, Andreu Altés, Jesús F. García‐Gavilán, Jadwiga Konieczna, J. Alfredo Martínez, Jordi Salas‐Salvadó

**Affiliations:** ^1^ Universitat Rovira i Virgili. Departament de Bioquímica i Biotecnologia, Alimentació, Nutrició, Desenvolupament i Salut Mental ANUT‐DSM Reus Spain; ^2^ Centro de Investigación Biomédica en Red Fisiopatología de la Obesidad y la Nutrición (CIBEROBN) Institute of Health Carlos III Madrid Spain; ^3^ Institut d'Investigació Sanitària Pere Virgili (IISPV), Reus Spain; ^4^ Department of Preventive Medicine and Public Health Instituto de Investigación Sanitaria de Navarra (IdiSNA), University of Navarra Pamplona Spain; ^5^ Research Group on Nutritional Epidemiology & Cardiovascular Physiopathology (NUTRECOR) Health Research Institute of the Balearic Islands (IdISBa) Palma de Mallorca Spain; ^6^ Department of Nutrition, Food Sciences and Physiology, Center for Nutrition Research, IdiSNA, Instituto de Nutrición y Salud (INS) University of Navarra Pamplona Spain; ^7^ Precision Nutrition and Cardiometabolic Health Program, IEA Food, CEI UAM + CSIC Madrid Spain; ^8^ Departamento de Medicina y Endocrinología Universidad de Valladolid Valladolid España; ^9^ Department of Internal Medicine, Institut d'Investigacions Biomèdiques August Pi Sunyer (IDIBAPS), Hospital Clinic University of Barcelona Barcelona Spain; ^10^ Institut de Recerca en Nutrició i Seguretat Alimentaria (INSA‐UB) University of Barcelona Barcelona Spain; ^11^ Centro de investigación Biomédica en Red de Epidemiología y Salud Pública (CIBERESP). Instituto de Salud Carlos III. Madrid, Spain; Institute of Biomedicine (IBIOMED) University of León León Spain; ^12^ CIBER Diabetes y Enfermedades Metabólicas (CIBERDEM), Instituto de Salud Carlos III (ISCIII) Madrid Spain; ^13^ Department of Endocrinology, Institut d'Investigacions Biomédiques August Pi Sunyer (IDIBAPS) Hospital Clinic, University of Barcelona Barcelona Spain; ^14^ Unit of Cardiovascular Risk and Nutrition Hospital del Mar Research Institute (IMIM) Barcelona Spain; ^15^ Osasunbidea, Servicio Navarro de Salud Pamplona Spain

**Keywords:** aging, cognitive decline, cognitive impairment, older adults, sarcopenic obesity

## Abstract

**Background:**

Sarcopenic obesity has been suggested as a potential risk factor for cognitive decline; however, few prospective studies have been conducted to test this hypothesis. We aimed to assess the relationship between baseline sarcopenic obesity and 6‐year trajectories of cognitive performance and subtle cognitive impairment in older adults.

**Methods:**

This longitudinal study comprised 1097 older adults aged 55–75 years (mean [SD] age, 65.3 [4.9] years; 506 females [46.1%] and 591 males [53.9%]) exhibiting baseline overweight/obesity and metabolic syndrome. Baseline lower‐limb muscle strength was determined by the validated 30‐s Chair Stand Test. Baseline total body weight, fat mass percentage and appendicular lean mass were obtained through dual‐energy x‐ray absorptiometry scans. Baseline sarcopenic obesity (*n* = 364 [33.2%]) was subsequently defined based on the new European Society for Clinical Nutrition and Metabolism and the European Association for the Study of Obesity consensus criteria. Cognitive performance was assessed at baseline, 2, 4 and 6 years of follow‐up through five composite scores derived from a comprehensive battery of eight validated neuropsychological tests, encompassing global cognitive function, general cognitive function, executive function, attention and language. Subtle cognitive impairment was defined for those *z*‐scores 0.5 standard deviations below the mean for each cognitive performance composite score at baseline. Linear and logistic two‐level mixed models including lost to follow‐up participants were fitted as main analyses. Complete case analyses were additionally performed.

**Results:**

After adjusting for multiple covariates, main analyses showed that, compared to older adults without sarcopenic obesity, those with sarcopenic obesity showed a higher decline in global cognitive function (between‐group difference, −1.0 [95% CI, −2.2 to 0.2] after 6 years; overall *p* = 0.048) and general cognitive function (between‐group difference, −2.5 [95% CI, −4.4 to 0.5] after 6 years; overall *p* = 0.028) and had a higher risk of subtle global cognitive function impairment (between‐group difference, 2.3 [95% CI, 0.9 to 5.6] after 6 years; overall *p* = 0.038) over 6 years of follow‐up. Associations remained consistent in the complete case analysis and attenuated when comparing those participants with baseline sarcopenic obesity with those only presenting sarcopenia, obesity or overweight. Of note, participants with baseline sarcopenia or obesity, compared to the absence of these conditions, showed no relationship with cognitive performance and subtle cognitive impairment over 6 years of follow‐up.

**Conclusions:**

Sarcopenic obesity may be considered a potential independent risk factor for cognitive decline over the long term that warrants further evaluation in clinical settings.

## 1 Introduction

Sarcopenic obesity, a condition combining sarcopenia and obesity [1], and cognitive impairment have emerged as major healthcare and public health concerns [2, 3], driven by population aging alongside rising rates of obesity, physical inactivity and unhealthy lifestyles [4, 5]. Sarcopenic obesity poses a dual threat to the physical and cognitive well‐being of older adults, heightening their dependence on healthcare services and imposing considerable socioeconomic burdens on health systems [6]. Consequently, there is a growing imperative for healthcare policymakers to address the potential implications of sarcopenic obesity on cognitive decline [7].

Evidence suggests that older adults with sarcopenic obesity tend to exhibit lower cognitive performance and a higher incidence of cognitive impairment compared to those without this condition [8]. Differences across studies and the potential difficulties in the interpretation and application of the findings [9] may be attributed to the multiple methodological approaches used in body composition measurements, cognitive assessment techniques through individual tests, rather than encompassing a more holistic approach using cognitive performance composite scores, population characteristics and usage of non‐standardised criteria for diagnosing sarcopenic obesity [10]. Of note, most of the studies presented a cross‐sectional design and used a wide variety of different criteria to classify individuals with or without sarcopenic obesity and cognitive performance [9, 10, 11, 12, 13] or cognitive impairment [14, 15, 16, 17].

The use of arbitrary cut‐offs for sarcopenic obesity and reliance on single‐test cognitive outcomes have hampered precise estimation of the impact of sarcopenic obesity on cognitive health, calling into question its value as a clinical predictor of cognitive decline and impairment [18]. Cognitive performance composite scores improve reliability, reduce error and increase power to detect subtle changes, especially in older adults, where single‐test scores are more variable [19, 20, 21]. To enhance comparability and clinical relevance, the new European Society for Clinical Nutrition and Metabolism (ESPEN) and the European Association for the Study of Obesity (EASO) consensus criteria, provide harmonised definitions and diagnostic thresholds for sarcopenic obesity and advocate for deeper research on sarcopenic obesity diagnostics as a relevant clinical priority and a future objective of sarcopenic obesity‐related comorbidities [1]. In a recent umbrella review of systematic reviews with meta‐analysis, it was acknowledged that sarcopenic obesity may be considered a potential risk factor for cognitive impairment, with evidence coming only from cross‐sectional and case–control studies, underscoring the need for longitudinal research using the aforementioned standardised frameworks and extended follow‐up to clarify cognitive trajectories [22].

To date, only one longitudinal study has addressed this research question only for cognitive performance, reporting a higher risk of cognitive performance decline over an 8‐year period in those individuals with sarcopenic obesity compared to those without this condition at baseline [23]. In addition, to the best of our knowledge, the association between sarcopenic obesity and risk of cognitive impairment has never been explored [24]. Therefore, the present study aims to investigate the association between baseline sarcopenic obesity and 6‐year trajectories of cognitive performance and subtle cognitive impairment in older adults with overweight or obesity and metabolic syndrome. By addressing key gaps in the literature, specifically by employing the newly established ESPEN‐EASO criteria for diagnosing sarcopenic obesity, focusing on domain‐specific cognitive performance outcomes and subtle cognitive impairment and utilising a long‐term longitudinal design, we hypothesised sarcopenic obesity to be associated with greater cognitive performance decline and an increased risk of subtle cognitive impairment over time compared to the absence of this condition.

## 2 Materials and Methods

### 2.1 Study Design

**FIGURE 1:**
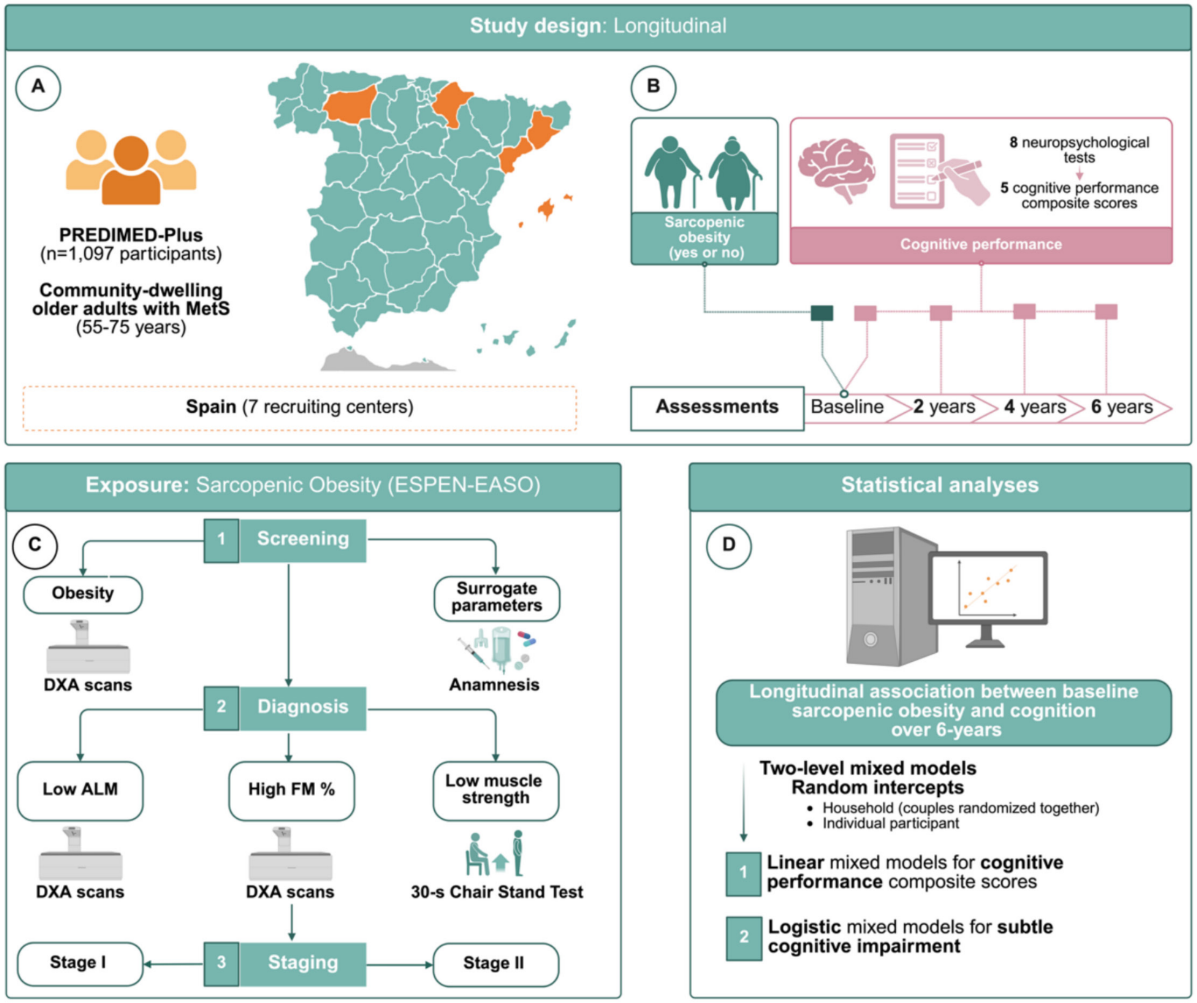
Study design. ALM, appendicular lean mass; DXA, dual‐energy x‐ray absorptiometry; FM, fat mass; MetS, metabolic syndrome; PREDIMED‐Plus, Prevención con Dieta Mediterránea–Plus.

The present study conducted an observational prospective study design within the framework of the PREDIMED‐Plus trial (Figure 1) [25]. Briefly, PREDIMED‐Plus is a multicentre, parallel‐group, randomised, single‐blind clinical trial evaluating the long‐term effects of a lifestyle intervention including an energy‐reduced Mediterranean diet, physical activity promotion and behavioural support for weight loss (intervention group) vs. general ad libitum Mediterranean diet recommendations (control group) on primary cardiovascular disease prevention and total body weight loss. Further details regarding the trial protocol can be accessed at https://www.predimedplus.com/ and in previously published sources [26, 27]. Ethical approval was obtained from all participating centres, and written informed consent was obtained from all participants. The trial was registered in 2014 at www.isrctn.com/ISRCTN89898870.

### 2.2 Participants

**FIGURE 2:**
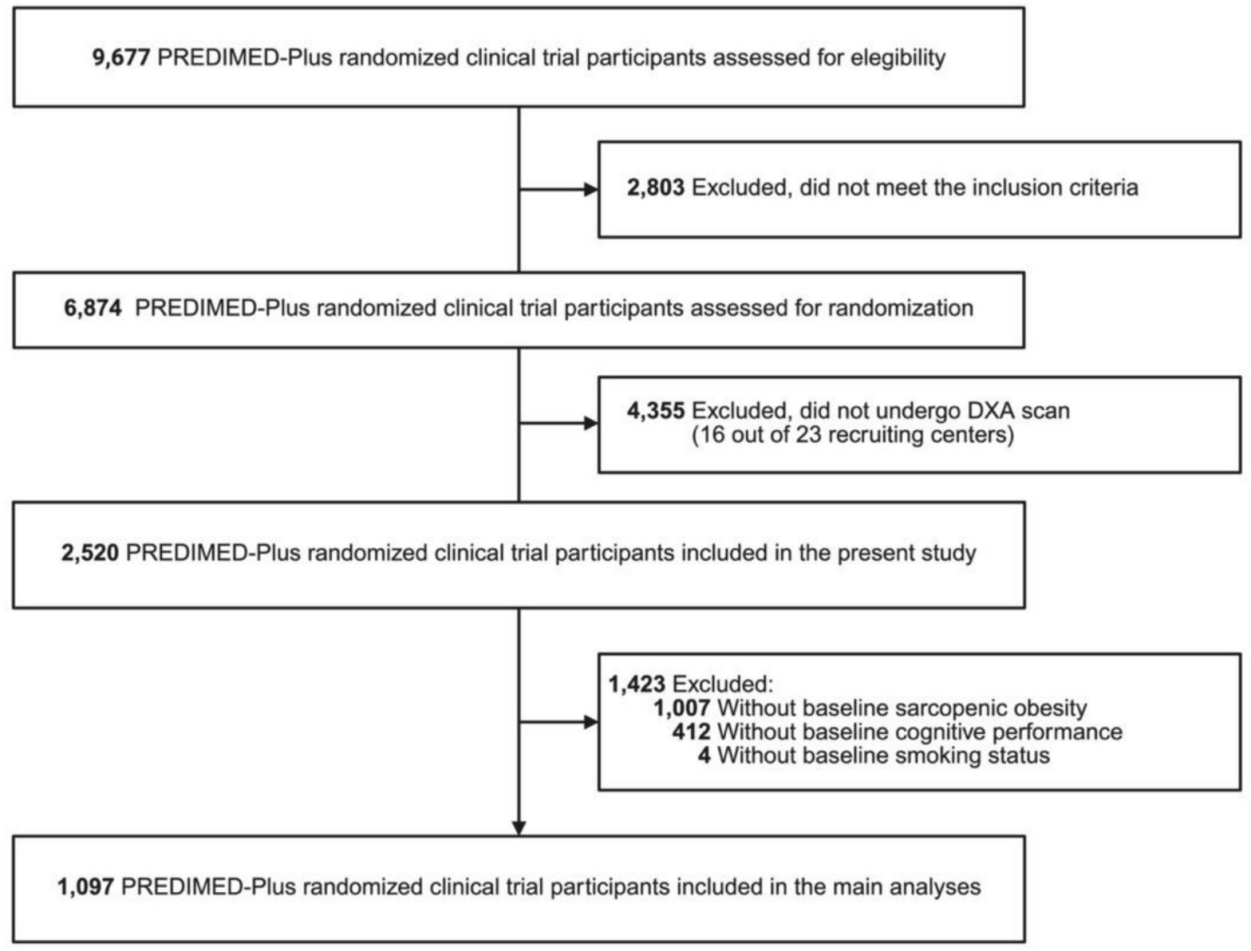
Flowchart of the study population. DXA, dual‐energy x‐ray absorptiometry; PREDIMED‐Plus, Prevención con Dieta Mediterránea–Plus.

The study enrolled community‐dwelling adults aged 55 to 75 years with overweight or obesity (body mass index [BMI] ranging from 27 to 40 kg/m^2^) who met at least three criteria for metabolic syndrome (Figure 1a) [28]. To identify metabolic syndrome, the following updated criteria from the International Diabetes Federation, the American Heart Association and the National Heart, Lung and Blood Institute were used: hypertension (≥ 130/85 mmHg), plasma triglycerides (≥ 150 mg/dL), plasma high‐density lipoprotein cholesterol (HDL [< 40 mg/dL for men or < 50 mg/dL for women]), fasting blood glucose (≥ 100 mg/dL) and central obesity (≥ 102 cm for men or ≥ 88 cm for women) [28]. Exclusion criteria were based on (I) unwillingness to give written informed consent, (II) institutionalisation, (III) pre‐existing cardiovascular diseases, psychiatric disorders or bowel diseases, (IV) weight loss medication use and (V) inability to follow the intervention based on religious purposes, food allergies or intolerances or injuries. From October 2013 to December 2016, a total of 6874 eligible participants were randomly assigned in a 1:1 ratio to either the intervention group or the control group. The randomisation procedure was blinded to all staff members and principal investigators. For participant couples sharing the same household, randomisation was done by cluster, with the couple as the unit of randomisation. For the purposes of the present study, data from a subsample of participants who underwent dual energy x‐ray absorptiometry (DXA) measurements in 7 of the 23 recruiting centres having access to DXA scanners (DXA sub study) were used. A total of 2520 participants were eligible for the present analysis, with 1097 participants presenting available information at baseline (Figure 2).

### 2.3 Sarcopenic Obesity Assessment

Lower‐limb muscle strength was determined at baseline using the validated 30‐s Chair Stand Test, based on the times participants stand and sit in a chair within 30 s, following an established protocol [1]. Height (cm) was measured at baseline in light clothing and without shoes with the use of a calibrated scale and a wall‐mounted stadiometer. Baseline dual‐energy x‐ray absorptiometry scans (Lunar iDXA and DXA Lunar Prodigy Primo, GE Healthcare, Madison, WI) were performed by trained radiology technicians to assess body composition following a validated standardised protocol and subject positioning provided by the manufacturer. The DXA was calibrated daily according to the manufacturer's guidelines. Total bone mineral content, total lean mass, total fat mass and regionally distributed lean mass in arms and legs (appendicular lean mass [ALM]) were obtained. Total body weight (kg) was calculated as the sum of total bone mineral content, total lean mass and total fat mass. ALM (kg) was calculated as the sum of the lean mass from the four limbs. BMI was calculated as total body weight (kg)/height (m)^2^). The ALM to total body weight ratio (ALM [kg]/total body weight [kg] × 100) was also determined.

According to the new criteria established by the ESPEN‐EASO consensus for Caucasian older adults, obesity was defined as a BMI ≥ 30 kg/m^2^ [1]. Low lower‐limb muscle strength was determined using the following age‐ and sex‐specific cut‐off points: 15 repetitions for females and 17 for males aged 55–64 years; 15 for females and 16 for males aged 65–69 years; 14 for females and 15 for males aged 70–74 years and 13 for females and 14 for males aged 75 years [1]. Low ALM was defined by the ALM to total body weight ratio, with diagnostic cut‐off points of < 28.27% for males and < 23.47% for females aged 55 years or older [1]. High fat mass percentage was defined using the following age‐ and sex‐specific thresholds: > 41% for females and > 29% for males aged 55–59 years, and > 43% for females and > 31% for males aged 60–75 years [1].

Baseline sarcopenic obesity was subsequently defined in accordance with the ESPEN‐EASO consensus as the simultaneous presence of obesity and surrogate biomarkers (screening criteria), in combination with both low muscle strength and concomitant low ALM and high fat mass percentage (diagnostic criteria). Individuals meeting these criteria were further stratified into two clinical stages based on the presence of complications (staging), aiming to reflect the severity of sarcopenic obesity and provide insight into potential primary or secondary forms. Stage I included participants with no complications attributable to altered body composition or skeletal muscle function, while Stage II comprised those with at least one such complication (e.g., metabolic diseases, disabilities related to high fat mass and/or low ALM, cardiovascular or respiratory conditions) (Figure 1c) [1].

### 2.4 Cognitive Performance and Subtle Cognitive Impairment Evaluation

Cognitive performance evaluation was conducted by trained personnel at baseline, 2, 4 and 6 years of follow‐up (Figure 1b). A battery of eight neuropsychological tests, validated specifically for the Spanish population, was administered via individual interviews. These tests comprised the Mini‐Mental State Examination, the Clock Drawing Test, the Verbal Fluency Tests, the forward and backward versions of the Digit Span Test (DST‐f and DST‐b, respectively) from the Wechsler Adult Intelligence Scale‐III and the Trail Making Test parts A and B (TMT‐A and TMT‐B). Detailed descriptions of these neuropsychological assessments can be found elsewhere [29]. Each cognitive test administered was standardised for every participant to a *z*‐score, utilising the mean and standard deviation (SD) derived from the baseline data [21, 30].

Composite measures for four cognitive composite scores (general cognitive function, executive function, attention and language), as well as a global assessment of cognitive function (GCF), were computed for each participant to delineate the domains of cognitive function that are likely to be affected based on standardised criteria [31]. Using cognitive performance composite scores reduces Type I error and improves sensitivity to subtle longitudinal changes by synthesising performance across multiple neuropsychological tests, which may fluctuate individually over time [20, 32]. The cognitive performance composite scores were determined by aggregating or deducting individual test *z*‐scores based on whether a higher score indicates superior or inferior cognitive performance, respectively, as detailed in Table S1. Following this procedure, the resulting five cognitive performance composite scores were further standardised to *z*‐scores using the mean and SD values from baseline cognitive performance composite score data. Older adults with baseline *z*‐scores 0.5 SD below the mean for each of the five cognitive performance composite scores were classified as presenting subtle cognitive impairment [33, 34]. This threshold was defined to identify participants with poorer cognitive performance with early cognitive difficulties that do not yet reach the threshold for mild cognitive impairment (MCI) [35].

### 2.5 Covariate Assessments

Sociodemographic and lifestyle information regarding age, sex, education level, marital status and smoking status was collected through administered questionnaires. Physical activity was estimated utilising the validated REGICOR (Registre Gironí del Cor) Short Physical Activity Questionnaire for the adult population adapted from the Minnesota Leisure Time Physical Activity Questionnaire [36], whereas sedentary behaviour was obtained using the validated Spanish version of the Nurses' Health Study questionnaire [37]. Personal medical history, encompassing conditions such as type 2 diabetes, hypercholesterolaemia, metabolic syndrome components (i.e., hypertension, hypertriglyceridaemia, low‐HDL cholesterol, hyperglycaemia and central obesity), as well as medication usage, was either self‐reported or extracted from medical records. Depressive symptomatology was evaluated using the Beck Depression Inventory‐II [38, 39]. Dietary intake was collected using a validated 143‐item Food Frequency Questionnaire [40]. Subsequently, daily energy intake and alcohol consumption estimations were derived utilising Spanish Food Composition Tables [41].

### 2.6 Statistical Analyses

Main analyses were performed in the evaluable population including all baseline available data for exposure, outcome and confounders and those participants lost to follow‐up over 2, 4 and 6 years of follow‐up.

Baseline characteristics of the study population were presented for both, overall participants and categorised by the presence of sarcopenic obesity, as means ± SDs for continuous variables and numbers (percentages) for categorical variables. Unpaired Student's t‐test and Chi‐square tests were employed for continuous and categorical variables, respectively.

Two‐level linear mixed models and two‐level logistic mixed models were conducted to assess the relationship between baseline sarcopenic obesity and cognitive performance composite scores and subtle cognitive impairment, respectively, over 2, 4 and 6 years, with random intercepts at cluster family (as couples from the same household were randomised together) and individual participants' levels (Figure 1d). In both cases, an interaction term between baseline sarcopenic obesity (yes or no) and time, age (years), sex (male or female) and recruiting centre was included as fixed effects in the basic models. Intervention group (control or intervention), baseline education level (primary or less, secondary or college), marital status (single, divorced or separated, married or widower), smoking status (current, former or never), depressive symptomatology (yes or no), type 2 diabetes prevalence (yes or no), hypertension prevalence (yes or no), hypercholesterolaemia prevalence (yes or no), physical activity (metabolic equivalents in minutes per day) and time varying sedentary time (hours per day), alcohol consumption (grams per day) and total energy intake (kilocalories per day) were additionally included as fixed effects in the multivariable‐adjusted models. Linear mixed models results were presented as intragroup mean changes and intergroup mean differences, whereas the results of logistic mixed models were presented as odds ratios (ORs), along with their corresponding 95% CIs.

A priori interaction analyses were conducted for cognitive performance composite scores by baseline categories of age (< 65 or ≥ 65 years), sex (male or female), intervention group (control or intervention), depressive symptomatology (yes or no), type 2 diabetes prevalence (yes or no), physical activity (< median or ≥ median), alcohol consumption (< median or ≥ median) and energy intake (< median or ≥ median), using the likelihood ratio test. An interaction term between time, sarcopenic obesity and each potential effect modifier was included within multivariable‐adjusted models.

Moreover, as sensitivity analyses, we (I) conducted a complete case analysis by excluding participants with missing cognitive performance data at 2, 4 and 6 years of follow‐up and (II) examined trajectories of cognitive performance and subtle cognitive impairment by comparing baseline sarcopenic obesity with sarcopenia‐only, obesity‐only or overweight‐only (i.e., without sarcopenia or obesity), as well as baseline sarcopenia‐only or obesity‐only with participants without these conditions.

All statistical analyses were conducted with Stata/SE version 14.2 (StataCorp LLC, College Station, TX, USA) using the PREDIMED‐Plus study dataset updated to December 19, 2023. All graphs were plotted using GraphPad Prism software v.9.0 (GraphPad Software, San Diego, CA, USA). Statistical significance was defined as a two‐tailed *p* < 0.05.

## 3 Results

Table 1 presents the baseline characteristics of the overall study population, stratified by the presence or absence of baseline sarcopenic obesity. A total of 1097 participants, with a mean age of 65.3 (4.9) years (506 [46.1%] female), were included in the current analysis. A total of 364 (33.2%) participants were identified as presenting sarcopenic obesity at baseline, all of whom were Caucasians and met the criteria for stage II of the condition as all of them presented metabolic syndrome. Compared with those without this condition, participants with baseline sarcopenic obesity presented higher age, depressive symptomatology scores, sedentary behaviour and lower educational level and physical activity (all *p* ≤ 0.041; Table 1). Lower baseline cognitive performance and higher subtle cognitive impairment were also observed for all domains (all *p* ≤ 0.035; Table 1). The numbers of study participants with and without data on sarcopenic obesity variables from centres having access to DXA devices are shown in Table S2. The baseline participant characteristics of those selected for DXA measurements compared with those not selected from the total cohort are shown in Table S3.

**TABLE 1:** Baseline characteristics of the PREDIMED‐Plus participants .

Characteristic	Total subsample of PREDIMED‐Plus (*n* = 1097)	Non sarcopenic obesity (*n* = 733)	Sarcopenic obesity (*n* = 364)	*p*
**Sociodemographic variables**				
Age, mean (SD), years	65.3 ± 4.9	65.1 ± 4.9	65.7 ± 4.8	**0.041**
Female, *n* (%)	506 (46.1)	323 (44.1)	183 (50.3)	0.052
Education level, *n* (%)				
Primary or less	501 (45.7)	309 (42.2)	192 (52.8)	**0.004**
Secondary	349 (31.8)	248 (33.8)	101 (27.8)	
College	247 (22.5)	176 (24.0)	71 (19.5)	
Civil status, *n* (%)				
Single, divorced or separated	146 (13.3)	90 (12.3)	56 (15.4)	0.056
Married	851 (77.6)	584 (79.7)	267 (73.4)	
Widower	100 (9.1)	59 (8.1)	41 (11.3)	
Ethnicity				
Caucasian	1097 (100)	733 (100)	364 (100)	—
African	0 (0)	0 (0)	0 (0)	
Asian	0 (0)	0 (0)	0 (0)	
Hispanic or Latino	0 (0)	0 (0)	0 (0)	
Other	0 (0)	0 (0)	0 (0)	
**Disease presence or medication usage at recruitment**				
Type 2 diabetes, *n* (%)	271 (24.7)	184 (25.1)	87 (23.9)	0.664
Hypercholesterolaemia, *n* (%)	742 (67.6)	495 (67.5)	247 (67.9)	0.913
Depression, *n* (%)	216 (19.7)	130 (17.7)	86 (23.6)	**0.021**
Metabolic syndrome, *n* (%)	1097 (100)	733 (100)	364 (100)	—
Hypertension, *n* (%)	924 (84.2)	614 (83.8)	310 (85.2)	0.549
Hypertriglyceridaemia, *n* (%)	435 (39.7)	302 (41.2)	133 (36.5)	0.137
Low HDL, *n* (%)	495 (45.1)	339 (46.3)	156 (42.9)	0.288
Hyperglycaemia, *n* (%)	711 (64.8)	467 (63.7)	244 (67.0)	0.278
Central obesity, *n* (%)	862 (78.6)	577 (78.7)	285 (78.3)	0.873
Medication use, *n* (%)				
Insulin or other antidiabetic drugs	209 (19.1)	135 (18.4)	74 (20.3)	0.448
Antihypertensive agents	889 (81.0)	585 (79.8)	304 (83.5)	0.140
Statins or other hypolipidaemic drugs	553 (50.4)	362 (49.4)	191 (52.5)	0.336
**Lifestyle variables**				
Physical activity, mean (SD), METs/min per day	402.9 ± 340.1	431.8 ± 346.2	344.9 ± 320.0	**0.001**
Sedentary time, mean (SD), h/d	5.8 ± 1.9	5.7 ± 1.8	6.0 ± 1.9	**0.005**
Smoking status, *n* (%)				
Current smoker	139 (12.7)	87 (11.9)	52 (14.3)	0.102
Former smoker	495 (45.1)	347 (47.3)	148 (40.7)	
Never smoker	463 (42.2)	299 (40.8)	164 (45.1)	
**Sarcopenic obesity variables**				
BMI, mean (SD), kg/m^2^	32.0 ± 3.3	31.0 ± 3.1	34.2 ± 2.7	**0.001**
Lower‐limb muscle strength, mean (SD), n	14.8 ± 4.8	16.2 ± 4.9	11.9 ± 2.7	**0.001**
ALM to total body weight ratio, mean (SD), %	25.5 ± 3.5	26.5 ± 3.4	23.6 ± 2.9	**0.001**
Total fat mass, mean (SD), %	40.1 ± 6.9	38.3 ± 6.8	43.6 ± 5.8	**0.001**
**Dietary variables**				
Alcohol intake, mean (SD), g/d	12.2 ± 15.7	12.2 ± 15.6	12.3 ± 15.8	0.954
Energy intake, mean (SD), kcal/d	2462 ± 560	2457 ± 575	2474 ± 528	0.628
**Cognitive performance**				
Global cognitive function	−0.1 ± 1.0	0.1 ± 1.0	−0.2 ± 1.0	**0.001**
*Cohen's d effect size*		0.24		
General cognitive function	0.1 ± 0.9	0.1 ± 0.9	−0.1 ± 1.0	**0.039**
*Cohen's d effect size*		0.13		
Executive function	0.1 ± 1.0	0.1 ± 1.0	−0.1 ± 1.0	**0.001**
*Cohen's d effect size*		0.23		
Attention	0.1 ± 1.0	0.1 ± 1.0	−0.1 ± 1.0	**0.001**
*Cohen's d effect size*		0.21		
Language	0.1 ± 1.0	0.2 ± 1.0	−0.1 ± 1.0	**0.002**
*Cohen's d effect size*		0.20		
**Subtle cognitive impairment**				
Global cognitive function, *n* (%)	319 (29.1)	194 (26.5)	125 (34.4)	**0.007**
General cognitive function, *n* (%)	329 (30.0)	204 (27.8)	125 (34.3)	**0.027**
Executive function, *n* (%)	319 (29.1)	190 (25.9)	129 (35.4)	**0.001**
Attention, *n* (%)	319 (29.1)	192 (26.2)	127 (34.9)	**0.003**
Language, *n* (%)	324 (29.5)	200 (27.3)	124 (34.0)	**0.020**

*Note:* Data are presented as n (%) or mean ± SD for categorical and continuous variables, respectively. Significant values (*p* < 0.05) between participants with or without sarcopenic obesity at baseline were highlighted in bold type.

Abbreviations: ALM, appendicular lean mass; BMI, body mass index; HDL, high‐density lipoprotein cholesterol; METs, metabolic equivalents; PREDIMED‐Plus, Prevención con Dieta Mediterránea–Plus.

Table 2 and Figure S1 display the longitudinal associations between baseline sarcopenic obesity and changes in cognitive performance composite scores over 6 years. After adjusting for multiple covariates, compared to participants without baseline sarcopenic obesity, those with sarcopenic obesity showed a higher decline in global cognitive function (between‐group difference, −1.0 [95% CI, −2.2 to 0.2] after 6 years; overall *p* = 0.048) and general cognitive function (between‐group difference, −2.5 [95% CI, −4.4 to 0.5] after 6 years; overall *p* = 0.028) over 6 years of follow‐up. Associations remained consistent in the complete case analysis (Table S4) but were attenuated when comparing participants with baseline sarcopenic obesity to those with sarcopenia‐only, obesity‐only or overweight (Table S5). Moreover, participants with baseline sarcopenia or obesity, compared with those without these conditions, showed no association with cognitive performance (Table S6), and similarly, no relationship was observed between sarcopenia‐only and participants without obesity (data not shown) over 6 years of follow‐up.

**TABLE 2:** Baseline sarcopenic obesity and cognitive performance over 2, 4 and 6 years of follow‐up^a^.

		Basic model				Multivariable‐adjusted model			
Variable		Changes in non‐sarcopenic obesity participants	Changes in sarcopenic obesity participants	Mean difference in changes	*p* ^c^	Changes in non‐sarcopenic obesity participants	Changes in sarcopenic obesity participants	Mean difference in changes	*p* ^c^
**Global cognitive function**									
Year 2 vs. baseline	Mean (95% CI)^b^	**0.6 (0.2 to 1.1)**	**1.2 (0.6 to 1.9)**	0.6 (−0.2 to 1.0)	**0.047**	**0.6 (0.2 to 1.1)**	**1.2 (0.6 to 1.9)**	0.6 (−0.2 to 1.0)	**0.048**
Year 4 vs. baseline	Mean (95% CI)^b^	**−1.0 (−1.5 to −0.5)**	**−0.6 (−1.4 to 0.1)**	0.4 (−0.5 to 1.3)		**−1.0 (−1.5 to −0.5)**	**−0.6 (−1.4 to 0.1)**	0.4 (−0.5 to 1.3)	
Year 6 vs. baseline	Mean (95% CI)^b^	**−2.3 (−3.0 to −1.6)**	**−3.3 (−4.3 to −2.3)**	−1.0 (−2.2 to 0.2)		**−2.3 (−3.0 to −1.6)**	**−3.3 (−4.3 to −2.3)**	−1.0 (−2.2 to 0.2)	
**General cognitive function**									
Year 2 vs. baseline	Mean (95% CI)^b^	**0.8 (0.1 to 1.5)**	1.0 (−0.1 to 2.0)	0.2 (−1.1 to 1.4)	**0.026**	**0.8 (0.1 to 1.5)**	1.0 (−0.1 to 2.0)	0.2 (−1.1 to 1.4)	**0.028**
Year 4 vs. baseline	Mean (95% CI)^b^	**−2.1 (−3.0 to −1.3)**	**−1.6 (−2.8 to −0.4)**	0.5 (−1.0 to 1.9)		**−2.1 (−3.0 to −1.3)**	**−1.6 (−2.8 to −0.4)**	0.5 (−1.0 to 1.9)	
Year 6 vs. baseline	Mean (95% CI)^b^	**−4.3 (−5.4 to −3.2)**	**−6.8 (−8.4 to −5.2)**	**−2.5 (−4.4 to −0.5)**		**−4.3 (−5.4 to −3.2)**	**−6.8 (−8.4 to −5.2)**	**−2.5 (−4.4 to −0.5)**	
**Executive function**									
Year 2 vs. baseline	Mean (95% CI)^b^	**0.7 (0.3 to 1.2)**	**1.4 (0.8 to 2.1)**	0.7 (−0.1 to 1.5)	0.346	**0.7 (0.2 to 1.1)**	**1.4 (0.7 to 2.0)**	0.7 (−0.1 to 1.5)	0.323
Year 4 vs. baseline	Mean (95% CI)^b^	−0.1 (−0.5 to 0.5)	0.2 (−0.5 to 1.0)	0.2 (−0.6 to 1.1)		−0.1 (−0.6 to 0.4)	0.1 (−0.6 to 0.9)	0.3 (−0.6 to 1.1)	
Year 6 vs. baseline	Mean (95% CI)^b^	**−1.2 (−1.7 to −0.7)**	**−1.0 (−1.7 to −0.2)**	−0.3 (−0.6 to 1.2)		**−1.1 (−1.7 to −0.6)**	**−1.1 (−1.9 to −0.3)**	0.1 (−0.9 to 1.0)	
**Attention**									
Year 2 vs. baseline	Mean (95% CI)^b^	−0.2 (−0.9 to 0.5)	0.2 (−0.8 to 1.2)	0.4 (−0.8 to 1.6)	0.389	−0.2 (−0.9 to 0.5)	0.2 (−0.8 to 1.1)	0.3 (−0.8 to 1.5)	0.598
Year 4 vs. baseline	Mean (95% CI)^b^	**−1.2 (−1.9 to −0.4)**	−0.7 (−1.8 to 0.4)	0.5 (−0.9 to 1.8)		**−1.2 (−2.0 to −0.4)**	−0.7 (−1.8 to 0.3)	0.5 (−0.8 to 1.8)	
Year 6 vs. baseline	Mean (95% CI)^b^	**−1.7 (−2.5 to −1.0)**	**−2.3 (−3.3 to −1.3)**	−0.6 (−1.9 to 0.7)		**−1.8 (−2.6 to −1.0)**	**−2.2 (−3.4 to −1.1)**	−0.5 (−1.9 to 1.0)	
**Language**									
Year 2 vs. baseline	Mean (95% CI)^b^	**0.9 (0.3 to 1.4)**	**1.4 (0.6 to 2.2)**	0.5 (−0.4 to 1.5)	0.201	**0.9 (0.3 to 1.4)**	**1.4 (0.6 to 2.2)**	0.5 (−0.4 to 1.5)	0.231
Year 4 vs. baseline	Mean (95% CI)^b^	**1.4 (0.8 to 2.0)**	**1.2 (0.3 to 2.1)**	−0.2 (−1.2 to 0.9)		**1.3 (0.7 to 2.0)**	**1.3 (0.4 to 2.2)**	−0.1 (−1.1 to 1.0)	
Year 6 vs. baseline	Mean (95% CI)^b^	0.5 (−0.1 to 1.1)	−0.1 (−1.0 to 0.8)	−0.6 (−1.6 to 0.5)		0.5 (−0.1 to 1.2)	−0.2 (−1.1 to 0.8)	−0.7 (−1.8 to 0.5)	

Abbreviation: CI, confidence interval.

^a^ Two‐level linear mixed models were fitted with random intercepts at cluster family (as couples from the same household were randomised together) and individual participants to assess relationships between the baseline presence of sarcopenic obesity (yes or no) (exposure) and cognitive function composite scores (outcome) measured repeatedly over time (at each follow‐up visit and for the overall follow‐up period). An interaction term between baseline sarcopenic obesity (yes or no) and time, age (years), sex (male or female) and recruiting centre were included as fixed effects in the basic models. Intervention group (control or intervention), baseline education level (primary or less, secondary or college), marital status (single, divorced or separated, married or widower), smoking status (current, former or never), depressive symptomatology (yes or no), type 2 diabetes prevalence (yes or no), hypertension prevalence (yes or no), hypercholesterolaemia prevalence (yes or no), physical activity (metabolic equivalents in minutes per day) and time varying sedentary time (hours per day), alcohol consumption (grams per day) and total energy intake (kilocalories per day) were additionally included as fixed effects in the multivariable‐adjusted models. Data are presented as mean standardised values (95% CI). Significant differences (*p* < 0.05) were highlighted in bold type. Overall (n = 1097), non‐sarcopenic obesity (n = 733) and sarcopenic obesity (n = 364).

^b^ Expressed as multiples of 10^−1^ (×10^−1^).

^c^ *p* values for the overall follow‐up period.

The following interactions were found for cognitive performance composite scores over 6 years by the presence or absence of baseline sarcopenic obesity (Figure S2): for global cognitive function and age (*p* = 0.028), sex (*p* = 0.027) and baseline energy intake (*p* = 0.002); for general cognitive function and baseline type 2 diabetes status (*p* = 0.028); for executive function and age (*p* = 0.001) and sex (*p* = 0.011); for attention and age (*p* = 0.019) and for language and age (*p* = 0.038) and baseline physical activity (*p* = 0.034). No significant interactions were observed for intervention group, depressive symptoms or alcohol intake. Additional stratified analyses by age and sex are presented in Tables S7 and S8, respectively.

Table 3 shows the relationship between baseline sarcopenic obesity and subtle cognitive impairment over 6 years. After adjusting for multiple confounders, compared to participants without sarcopenic obesity, those with baseline sarcopenic obesity presented a higher risk of subtle global cognitive function impairment (between‐group difference, 2.3 [95% CI, 0.9 to 5.6] after 6 years; overall *p* = 0.038) over 6 years of follow‐up. Results remained consistent in the complete case analysis (Table S9) and were attenuated when comparing participants with baseline sarcopenic obesity to those with sarcopenia or obesity or overweight alone (Table S10). Furthermore, no associations were observed between baseline sarcopenia or obesity alone and participants without these conditions (Table S11), nor between sarcopenia‐only and participants without obesity (data not shown), in relation to subtle cognitive impairment over 6 years.

**TABLE 3:** Baseline sarcopenic obesity and subtle cognitive impairment over 2, 4 and 6 years of follow‐up^a^.

Variable	Basic model		Multivariable‐adjusted model	
	Sarcopenic obesity	Overall *p* ^b^	Sarcopenic obesity	Overall *p* ^b^
**Global cognitive function**				
Year 2 vs. baseline	0.6 (0.3–1.1)	0.055	0.6 (0.3–1.1)	**0.038**
Year 4 vs. baseline	0.9 (0.5–1.8)		0.9 (0.4–1.7)	
Year 6 vs. baseline	2.1 (0.9–5.3)		2.3 (0.9–5.6)	
**General cognitive function**				
Year 2 vs. baseline	0.8 (0.5–1.2)	0.664	0.8 (0.5–1.2)	0.621
Year 4 vs. baseline	0.9 (0.6–1.4)		0.9 (0.6–1.4)	
Year 6 vs. baseline	1.1 (0.6–1.9)		1.1 (0.6–1.9)	
**Executive function**				
Year 2 vs. baseline	0.7 (0.4–1.2)	**0.044**	0.7 (0.4–1.2)	0.160
Year 4 vs. baseline	1.3 (0.7–2.5)		1.2 (0.6–2.3)	
Year 6 vs. baseline	1.7 (0.9–3.3)		1.5 (0.7–3.0)	
**Attention**				
Year 2 vs. baseline	1.1 (0.7–1.9)	0.957	1.1 (0.7–1.9)	0.864
Year 4 vs. baseline	1.0 (0.6–1.8)		1.0 (0.6–1.8)	
Year 6 vs. baseline	1.1 (0.7–1.9)		1.3 (0.7–2.3)	
**Language**				
Year 2 vs. baseline	0.9 (0.5–1.4)	0.801	0.8 (0.5–1.4)	0.768
Year 4 vs. baseline	0.9 (0.5–1.5)		0.8 (0.5–1.4)	
Year 6 vs. baseline	1.1 (0.6–1.9)		1.0 (0.6–1.9)	

Abbreviations: CI, confidence interval; OR, odds ratio; SD, standard deviation.

^a^ Two‐level logistic mixed models were fitted with random intercepts at cluster family (as couples from the same household were randomised together) and individual participants to assess relationships between the baseline presence of sarcopenic obesity (yes or no) (exposure) and subtle cognitive impairment (outcome) measured repeatedly over time (at each follow‐up visit and for the overall follow‐up period). An interaction term between baseline sarcopenic obesity (yes or no) and time, age (years), sex (male or female) and recruiting centre were included as fixed effects in the basic models. Intervention group (control or intervention), baseline education level (primary or less, secondary or college), marital status (single, divorced or separated, married or widower), smoking status (current, former or never), depressive symptomatology (yes or no), type 2 diabetes prevalence (yes or no), hypertension prevalence (yes or no), hypercholesterolaemia prevalence (yes or no), physical activity (metabolic equivalents in minutes per day) and time varying sedentary time (hours per day), alcohol consumption (grams per day) and total energy intake (kilocalories per day) were additionally included as fixed effects in the multivariable‐adjusted models. The non‐presence of sarcopenic obesity was considered as 1 (reference). Subtle cognitive impairment was defined as presenting a cognitive performance composite score less than or equal to the baseline 0.5 SD of the distribution for each *z*‐score. Data are presented as OR standardised values (95% CI). Overall (n = 1097), non‐sarcopenic obesity (n = 733) and sarcopenic obesity (n = 364).

^b^ *p* values for the overall follow‐up period.

## 4 Discussion

The present longitudinal research provides significant and novel prospective findings on the relationship between sarcopenic obesity and cognition using standardised criteria, as older adults with baseline sarcopenic obesity showed a greater cognitive performance decline and higher odds of subtle cognitive impairment over 6 years of follow‐up compared to those without sarcopenic obesity. In particular, after adjusting for multiple confounders, higher global and general cognitive performance decline and higher risk of subtle global cognitive function impairment were observed in older adults with baseline sarcopenic obesity compared to those without this condition but were attenuated when the non‐presence of sarcopenic obesity was further stratified into sarcopenia, obesity or overweight. Notably, sarcopenia or obesity alone, compared with the absence of these conditions, was not associated with cognitive decline or subtle cognitive impairment. Taken together, these findings deepen research on sarcopenic obesity diagnostics as a potential relevant clinical priority.

The aforementioned findings are consistent with previous studies examining the association between sarcopenic obesity and cognitive performance or impairment. Despite the heterogeneity in the criteria used to define both sarcopenic obesity and cognitive outcomes across cross‐sectional and case–control study designs, the evidence collectively suggests a link between sarcopenic obesity and cognitive decline or impairment [22]. Similarly, the unique longitudinal study to date addressing this research question defined sarcopenic obesity using the older consensus definitions of the Foundation of the National Institutes of Health [23, 42]. In this study, Batsis and colleagues also reported sarcopenic obesity to be associated with greater long‐term risk of impaired cognitive performance over the course of the 8‐year study in a sample of cognitively unimpaired older adults. Interestingly, similar associations were observed for the sarcopenia‐only group. However, their study included a larger sample size and a more balanced distribution of subgroups, which may have provided greater statistical power to detect these associations compared to our sarcopenia‐only analyses. It is also worth noting that cognitive performance was based on individual tests and not on composite scores. Moreover, the authors agreed as a clear limitation not to have classified sarcopenic obesity using the new ESPEN‐EASO consensus criteria [1, 23].

In our study, we additionally observed that the older participants with sarcopenic obesity presented a higher tendency to cognitive decline compared to younger individuals with the same condition. Of note, in the unique cross‐sectional study using the new ESPEN‐EASO consensus criteria, as used in the present study, the association between sarcopenic obesity and lower cognitive performance was significant only in those participants aged ≥ 60. These results may be due to the use of self‐reported memory questions or to the fact that older individuals are at higher risk of cognitive impairment [9]. In this context, although cognitive decline is a natural process associated with aging, its progression may be more accelerated in certain populations, such as in the case of older adults presenting sarcopenic obesity [43]. In parallel, we observed male participants with sarcopenic obesity to exhibit a higher risk of cognitive decline compared to their female counterparts. A cross‐sectional study investigating sex differences in the association between sarcopenic obesity and cognitive decline reported that sex‐related differences may be partially explained by the limited sample size, particularly among males, as women tend to exhibit a higher prevalence of sarcopenic obesity [13]. Despite sarcopenia or obesity independently, not being associated with cognitive performance decline and subtle cognitive impairment, it is important to note that the findings reported in our study may have been partially attenuated by the fact that older adults without sarcopenic obesity at baseline still exhibited metabolic syndrome and an unfavourable body composition profile. This underscores the potential impact of sarcopenic obesity itself as a more independent determinant of cognitive health, at least, in our cohort [22].

Understanding the underlying mechanisms behind the association between sarcopenic obesity and cognition is key for preventing this condition in at‐risk individuals [12]. Although the link is complex, sarcopenic obesity has been associated with insulin resistance and chronic inflammation, which are well‐known contributors to cognitive decline and impairment [44]. An increase in insulin resistance has been hypothesised to contribute to β‐amyloid accumulation in the brain, potentially increasing the risk of cognitive decline and impairment [45], whereas chronic inflammation markers such as C‐reactive protein, interleukin‐6 and tumour necrosis factor‐α have been correlated with a higher risk of cognitive decline and have been positively correlated with fat mass and negatively associated with ALM [11]. Individuals with sarcopenic obesity also present decreased growth hormone secretion, which is suggested to be involved in muscle strength and cognitive function preservation [14]. Furthermore, altered myokine and adipokine signalling in sarcopenic obesity may influence muscle–brain communication and the blood–brain barrier [24]. These pathways remain hypothetical, and future studies incorporating relevant biomarkers are needed to clarify the biological mechanisms linking sarcopenic obesity and cognitive outcomes.

Collectively, our findings underscore the importance of evaluating sarcopenic obesity in older adults as a feasible approach to potentially predict or mitigate cognitive performance decline or impairment as an independent and synergistic factor of sarcopenia and obesity. Further prospective studies aimed at determining whether sarcopenic obesity is a simple correlate of cognitive performance or has a role in the processes that lead to cognitive impairment are therefore warranted [12]. This analysis highlights the need to identify individuals with sarcopenic obesity in clinical practice. Multicomponent interventions targeting this condition are needed to reduce dementia risk and functional decline, potentially preventing institutionalisation. Future research should also assess cognitive decline and healthcare resource use in this population, with early interventions, even nonpharmacological, being potentially critical [23].

Our study presents notable strengths. First, its longitudinal prospective design facilitated the observation of temporal associations over a 6‐year follow‐up period, although this design does not establish potential causal relationships. Second, the comprehensive assessment of cognitive performance utilised a diverse array of neuropsychological tests, enabling the measurement of composite scores across multiple cognitive performance domains. Lastly, the study benefited from a large sample size, affording the adjustment of statistical models for various potential confounding factors. Nevertheless, our study findings should be interpreted in light of certain limitations. Firstly, the potential for reverse causality and residual confounding persists, particularly from unmeasured factors not accounted for in the analyses. Secondly, the generalizability of the results may be limited to older populations with overweight/obesity and metabolic syndrome, precluding extrapolation to other populations. Thirdly, ALM and fat mass are mathematically intertwined in DXA measurements, making it difficult to fully separate the independent effects of muscle and adiposity on cognitive outcomes. Of note, we used DXA‐derived ALM, which, although limb‐focused, remains a proxy that includes non‐muscle tissues and extracellular water rather than direct skeletal muscle. This may attenuate true muscle–outcome associations and inflate independent effects of adiposity. Studies using direct muscle measures (e.g., D_3_‐creatine dilution) report stronger associations and minimal added effect of adiposity, supporting the use of direct methods to more accurately quantify skeletal muscle and its health effects [46]. Fourthly, the sample size of the non‐sarcopenic obesity subgroups is relatively small, especially for the sarcopenia‐only group, which may limit statistical power when the non‐sarcopenic obesity group is divided into three separate subgroups. Fifthly, although the observed differences in cognitive scores were statistically significant, a longer follow‐up might have revealed larger or more clinically meaningful differences. Sixthly, as PREDIMED‐Plus is a randomised controlled trial, although all the analyses were adjusted for the intervention group and showed no a priori interaction, the lifestyle advice that participants received could be affecting our findings [27]. Of note, dietary patterns and physical activity have a direct impact on body weight that is also critical, as overweight or obesity are linked to cognitive decline [47].

## 5 Conclusion

In conclusion, the findings of this study highlight that sarcopenic obesity together with clinical complications may be a significant and potential independent risk factor for cognitive performance decline and subtle cognitive impairment over the long term. These results underscore the importance of evaluating sarcopenic obesity in clinical settings as a potential predictor and risk factor for future cognitive decline. Future research should focus on standardising diagnostic criteria for sarcopenic obesity, exploring potential underlying mechanisms and refining interventions taking into account the potential moderating effects of age and sex, with the aim of mitigating concomitant sarcopenic obesity and cognitive decline.

## Author Contributions

**Héctor Vázquez‐Lorente:** writing—original draft, writing—review and editing, visualisation, methodology, investigation, data curation, conceptualisation. **Indira Paz‐Graniel:** writing—review and editing, methodology, formal analysis, data curation, conceptualisation. **Hernando J. Margara‐Escudero:** writing—review and editing, visualisation, methodology, formal analysis, data curation, conceptualisation. **Miguel Ángel Martínez‐González:** writing—review and editing, investigation, data curation, conceptualisation, funding acquisition. **Dora Romaguera:** writing—review and editing, investigation, data curation, conceptualisation, funding acquisition. **D. Martinez Urbistondo:** writing—review and editing. **Ramon Estruch:** writing—review and editing, investigation, data curation, conceptualisation, funding acquisition. **Vicente Martín Sánchez:** writing—review and editing, investigation, data curation, conceptualisation. **Josep Vidal:** writing—review and editing, investigation, data curation, conceptualisation. **Montserrat Fitó:** writing—review and editing, investigation, data curation, conceptualisation. **Nuria Goñi:** writing—review and editing. **Alice Chaplin:** writing—review and editing. **M. Angeles Zulet:** writing—review and editing. **Emilio Sacanella:** writing—review and editing. **José Antonio de Paz Fernández:** writing—review and editing. **Andreu Altés:** writing—review and editing. **Jesús F. García‐Gavilán:** writing—review and editing, methodology, formal analysis, data curation, conceptualisation. **Jadwiga Konieczna:** writing—review and editing, data curation, funding acquisition. **J. Alfredo Martínez:** writing—review and editing, investigation, data curation, conceptualisation, funding acquisition. **Jordi Salas‐Salvadó:** writing—original draft, writing—review and editing, visualisation, validation, methodology, investigation, data curation, conceptualisation, funding acquisition.

## Funding

This study was supported by the official Spanish Institutions for Funding Scientific Biomedical Research, CIBER Fisiopatología de la Obesidad y Nutrición (CIBEROBN) and Instituto de Salud Carlos III (ISCIII), through the Fondo de Investigación para la Salud (FIS), which is co‐funded by the European Regional Development Fund (six coordinated FIS projects led by J.S.‐S. and J.V., including the following projects: PI13/00673, PI13/00492, PI13/00272, PI13/01123, PI13/00462, PI13/00233, PI13/02184, PI13/00728, PI13/01090, PI13/01056, PI14/01722, PI14/00636, PI14/00618, PI14/00696, PI14/01206, PI14/01919, PI14/00853, PI14/01374, PI14/00972, PI14/00728, PI14/01471, PI16/00473, PI16/00662, PI16/01873, PI16/01094, PI16/00501, PI16/00533, PI16/00381, PI16/00366, PI16/01522, PI16/01120, PI17/00764, PI17/01183, PI17/00855, PI17/01347, PI17/00525, PI17/01827, PI17/00532, PI17/00215, PI17/01441, PI17/00508, PI17/01732, PI17/00926, PI19/00957, PI19/00386, PI19/00309, PI19/01032, PI19/00576, PI19/00017, PI19/01226, PI19/00781, PI19/01560, PI19/01332, PI20/01802, PI20/00138, PI20/01532, PI20/00456, PI20/00339, PI20/00557, PI20/00886, PI20/01158 and PI23/00220); the Especial Action Project entitled: Implementación y evaluación de una intervención intensiva sobre la actividad física Cohorte PREDIMED‐Plus grant to J.S.‐S.; the European Research Council (Advanced Research Grant 2014–2019; agreement #340918) granted to M.A.M.‐G.; the Agencia Catalana de Recerca i Universitats, AGAUR (2021SGR00336 to J.S.‐S; the Recercaixa (number 2013ACUP00194) grant to J.S.‐S.; grants from the Consejería de Salud de la Junta de Andalucía (PI0458/2013, PS0358/2016 and PI0137/2018); the PROMETEO/21/2021 and the AICO/2021/347 grants from the Generalitat Valenciana; the MINECO (CNS2022–135862) grant to D.R. and the Horizon 2020 PRIME study (Prevention and Remediation of Insulin Multimorbidity in Europe; grant agreement #847879). J.K. received funding from ISCIII, through the Miguel Servet Project (CP24/00089), co‐funded by the European Union. H.V.‐L. was funded by a research grant from the Agència de Gestió d‘Ajuts Universitaris de Recerca via Unión Europea, Next Generation EU (AGAUR, record number: 2023POST‐INV‐01).H.J.M.‐E holds a Contrato Pre‐doctoral de Formacion en Investigacion en Salud (PFIS FI24/00038) funded by the ISCIII. None of the funding sources took part in the design, collection, analysis, interpretation of the data or writing of the report or in the decision to submit the manuscript for publication. J.S.‐S., senior author, gratefully acknowledges the financial support by ICREA under the ICREA Academia program.

## Ethics Statement

The study protocol was approved by the Research Ethics Committees of all recruiting centres. In addition, all participants signed an informed consent form upon entry into the study.

## Conflicts of Interest

The authors declare no conflicts of interest.

## Supporting information


**Table S1:** Composite cognitive assessment equations^a^.
**Table S2:** Number of study participants with and without data on sarcopenic obesity variables from centres having access to DXA device^a^.
**Table S3:** Baseline characteristics of study participants selected and non‐selected for DXA measurements^a^.
**Table S4:** Baseline sarcopenic obesity and cognitive performance over 2, 4 and 6 years of follow‐up after excluding lost to follow‐up participants (complete case analysis)^a^.
**Table S5:** Baseline sarcopenic obesity compared with sarcopenia or obesity or overweight alone and cognitive performance over 2, 4 and 6 years of follow‐up^a^.
**Table S6:** Baseline sarcopenia or obesity alone, compared with participants without these conditions, and cognitive performance over 2, 4 and 6 years of follow‐up^a^.
**Table S7:** Baseline sarcopenic obesity and cognitive performance over 2, 4 and 6 years of follow‐up by age^a^.
**Table S8:** Baseline sarcopenic obesity and cognitive performance over 2, 4 and 6 years of follow‐up by sex^a^.
**Table S9:** Baseline sarcopenic obesity and subtle cognitive impairment over 2, 4 and 6 years after excluding lost to follow‐up participants (complete case analysis)^a^.
**Table S10:** Baseline sarcopenic obesity compared with sarcopenia or obesity or overweight alone and subtle cognitive impairment over 2, 4 and 6 years of follow‐up participants^a^.
**Table S11:** Baseline sarcopenia or obesity compared with participants without these conditions and subtle cognitive impairment over 2, 4 and 6 years of follow‐up^a^.
**Figure S1:** Adjusted means for baseline sarcopenic obesity and cognitive performance over 2, 4 and 6 years of follow‐up. Data are presented as means (95% CI). Two‐level linear mixed models were fitted with random intercepts at cluster family (as couples from the same household were randomised together), and individual participants to assess relationships between the baseline presence of sarcopenic obesity (yes or no) (exposure) and cognitive function composite scores (outcome) measured repeatedly over time (at each follow‐up visit and for the overall follow‐up period). An interaction term between baseline sarcopenic obesity (yes or no) and time, age (years), sex (male or female) and recruiting centre were included as fixed effects in the basic models. Intervention group (control or intervention), baseline education level (primary or less, secondary or college), marital status (single, divorced or separated, married or widower), smoking status (current, former or never), depressive symptomatology (yes or no), type 2 diabetes prevalence (yes or no), hypertension prevalence (yes or no), hypercholesterolaemia prevalence (yes or no), physical activity (metabolic equivalents in minutes per day) and time varying sedentary time (hours per day), alcohol consumption (grams per day) and total energy intake (kilocalories per day) were additionally included as fixed effects in the multivariable‐adjusted models. CI, confidence interval.
**Figure S2:** A priory interaction for cognitive performance composite scores over 6 years by presence of baseline sarcopenic obesity with multiple baseline variables of the study. Two‐level linear mixed models were fitted with random intercepts at cluster family (as couples from the same household were randomised together), and individual participants to assess relationships between the baseline presence of sarcopenic obesity (yes or no) (exposure) and cognitive function composite scores (outcome) measured repeatedly over time (at each follow‐up visit and for the overall follow‐up period). An interaction term between baseline sarcopenic obesity (yes or no) and time, age (years) and sex (male or female) was included as fixed effects in the basic models. Intervention group (control or intervention), baseline education level (primary or less, secondary or college), marital status (single, divorced or separated, married or widower), smoking status (current, former or never), depressive symptomatology (yes or no), type 2 diabetes prevalence (yes or no), hypertension prevalence (yes or no), hypercholesterolaemia prevalence (yes or no), physical activity (metabolic equivalents in minutes per day) and time varying sedentary time (hours per day), alcohol consumption (grams per day) and total energy intake (kilocalories per day) were additionally included as fixed effects in the multivariable‐adjusted models. Data are presented as mean standardised values (95% CI). Significant values (*p* < 0.05) were highlighted in bold type. CI, confidence interval.

## Data Availability

Data described in the manuscript, codebook and analytic code will be made available upon request pending application and approval of the PREDIMED‐Plus Steering Committee. There are restrictions on the availability of data for the PREDIMED‐Plus trial, due to the signed consent agreements around data sharing, which only allow access to external researchers for studies following the project purposes. Requestors wishing to access the PREDIMED‐Plus trial data used in this study can make a request to the PREDIMED‐Plus trial Steering Committee chair: jordi.salas@urv.cat. The request will then be passed to members of the PREDIMED‐Plus Steering Committee for deliberation.
