# Supplementary material for: Sarcopenic Obesity and Longitudinal Trajectories on Cognitive Performance and Subtle Cognitive Impairment Over 6 Years in Older Adults
Source: J Cachexia Sarcopenia Muscle. 2025 Dec 8;16(6):e70158. doi: 10.1002/jcsm.70158 (PMC12685615; doi:10.1002/jcsm.70158)
Supplement: Supplementary file 1 — Table S1: Composite cognitive assessment equationsa. Table S2: Number of study participants with and without data on sarcopenic obesity variables from centres having access to DXA devicea. Table S3: Baseline characteristics of study participants selected and non‐selected for DXA measurementsa. Table S4: Baseline sarcopenic obesity and cognitive performance over 2, 4 and 6 years of follow‐up after excluding lost to follow‐up participants (complete case analysis)a. Table S5: Baseline sarcopenic obesity compared with sarcopenia or obesity or overweight alone and cognitive performance over 2, 4 and 6 years of follow‐upa. Table S6: Baseline sarcopenia or obesity alone, compared with participants without these conditions, and cognitive performance over 2, 4 and 6 years of follow‐upa. Table S7: Baseline sarcopenic obesity and cognitive performance over 2, 4 and 6 years of follow‐up by agea. Table S8: Baseline sarcopenic obesity and cognitive performance over 2, 4 and 6 years of follow‐up by sexa. Table S9: Baseline sarcopenic obesity and subtle cognitive impairment over 2, 4 and 6 years after excluding lost to follow‐up participants (complete case analysis)a. Table S10: Baseline sarcopenic obesity compared with sarcopenia or obesity or overweight alone and subtle cognitive impairment over 2, 4 and 6 years of follow‐up participantsa. Table S11: Baseline sarcopenia or obesity compared with participants without these conditions and subtle cognitive impairment over 2, 4 and 6 years of follow‐upa. Figure S1: Adjusted means for baseline sarcopenic obesity and cognitive performance over 2, 4 and 6 years of follow‐up. Data are presented as means (95% CI). Two‐level linear mixed models were fitted with random intercepts at cluster family (as couples from the same household were randomised together), and individual participants to assess relationships between the baseline presence of sarcopenic obesity (yes or no) (exposure) and cognitive function composite scores (outcome) measured [file JCSM-16-e70158-s001.docx]

| **Table S1.-** Composite cognitive assessment equations^a^ | |
| --- | --- |
| **Composite cognitive domain** | **Composite component score** |
| *Global Cognitive Function^b^* | = $\frac{zMMSE + zCDT+ zVFT-a + zVFT-p + \left( -zTMT-A \right)+ \left( -zTMT-B \right)+ zDST-f+ zDST-b}{8}$ |
| *General Cognitive Function^c^* | = $\frac{zMMSE + zCDT}{2}$ |
| *Executive Function^d^* | = $\frac{zVFT-a + zVFT-p + \left( -zTMT-B \right) + zDST-b}{4}$ |
| *Attention^e^* | = $\frac{\left( -zTMT-A \right)+ zDST-f}{2}$ |
| *Language^f^* | = $\frac{zVFT-a + zVFT-p}{2}$ |
| Abbreviations: CDT, Clock Drawing Test; DST-b, Digit Span test - backward; DST-f, Digit Span test - forward; MMSE, Mini-Mental State Examination; TMT-A, Trail Making Test Part A; TMT-B, Trail Making Test Part B; VFT-a, Verbal Fluency tasks semantical; VFT-p, Verbal Fluency tasks phonological.  ^a^Standardized scores of the TMT-A and TMT-B were inverted, so that higher scores would represent better cognitive function.  ^b^The global cognitive function was determined by computing the mean standardized individual scores of all neuropsychological tests.  ^c^The general cognitive function composite included the MMSE score and the CDT score.  ^d^The executive function composite included the mean standardized individual scores of the VFT-a score, VFT-p score, TMT-B score, and DST-b score.  ^e^The attention composite included the mean standardized individual scores of the TMT-A score and the DST-f score.  ^f^The language composite included the mean standardized individual scores of the VFT-a score and the VFT-p score. | |

| **Table S2.- Number of study participants with and without data on sarcopenic obesity variables from centers having access to DXA device^a^** | | | |
| --- | --- | --- | --- |
| **Variable** | **Total** | **Included^b^** | **Non-included^c^** |
|  | **n** | **n (%)** | **n (%)** |
| **Recruiting center** |  |  |  |
| All centers with available DXA | 2,520 | 1,513 (60.0) | 1,007 (40.0) |
| Center 3 | 331 | 261 (78.9) | 70 (21.1) |
| Center 4 | 335 | 297 (88.7) | 38 (11.3) |
| Center 5 | 303 | 138 (45.5) | 165 (54.5) |
| Center 7 | 460 | 352 (76.5) | 108 (23.5) |
| Center 10^d^ | 628 | 95 (15.1) | 533 (84.9) |
| Center 20 | 205 | 133 (64.9) | 72 (35.1) |
| Center 23 | 258 | 237 (91.9) | 21 (8.1) |
| Abbreviations: DXA, dual energy x-ray absorptiometry.  ^a^Data are shown as numbers (percentages). The sample of 1,513 participants come from 7 out of the 23 PREDIMED-Plus recruiting centers, as those seven centers were the only centers that had DXA scanner available. In each of these seven centers, either all participants or a sub-sample were invited to DXA scans.  ^b^Participants with data on body composition measured with DXA and included in the analysis.  ^c^Participants without DXA data excluded from the analysis.  ^d^Center #10 was the vanguard center and started the recruitment and randomization many months earlier than the other centers, therefore many participants of center #10 had had their baseline evaluation before the DXA study started. | | | |

| **Table S3.- Baseline characteristics of study participants selected and non-selected for DXA measurements^a^** | | | | |
| --- | --- | --- | --- | --- |
| **Characteristic** | **Total population (n=4,679)^d^** | **Selected^b^ (n=1,709)^d^** | **Non selected^c^ (n=2,970)^d^** | **P-value** |
| **Sociodemographic variables** |  |  |  |  |
| Age, mean (SD), years | 64.9 ± 4.9 | 65.4 ± 5.0 | 64.6 ± 4.8 | **0.001** |
| Female, No. (%) | 2,249 (48.0) | 788 (46.1) | 1,461 (49.2) | **0.042** |
| Education level, No. (%) |  |  |  |  |
| Primary or less | 2,241 (47.9) | 789 (46.2) | 1,452 (48.9) | 0.122 |
| Secondary | 1,384 (29.6) | 534 (31.2) | 850 (28.6) |  |
| College | 1,054 (22.5) | 386 (22.6) | 668 (22.5) |  |
| Civil status, No. (%) |  |  |  |  |
| Single, divorced or separated | 595 (12.7) | 220 (12.9) | 375 (12.6) | 0.889 |
| Married | 3,599 (76.9) | 1,308 (76.5) | 2,291 (77.1) |  |
| Widower | 485 (10.4) | 181 (10.6) | 304 (10.2) |  |
| Ethnicity |  |  |  |  |
| Caucasian | 4,679 (100) | 1,709 (100) | 2,970 (100) | – |
| African | 0 (0) | 0 (0) | 0 (0) |  |
| Asian | 0 (0) | 0 (0) | 0 (0) |  |
| Hispanic or Latino | 0 (0) | 0 (0) | 0 (0) |  |
| Other | 0 (0) | 0 (0) | 0 (0) |  |
| **Disease presence or medication usage at recruitment** | | |  |  |
| Type 2 diabetes, No. (%) | 1,333 (28.5) | 419 (24.5) | 914 (30.8) | **0.001** |
| Hypercholesterolemia, No. (%) | 3,256 (69.6) | 1,123 (65.7) | 2,133 (71.8) | **0.001** |
| Depression, No. (%) | 916 (19.6) | 324 (19.0) | 592 (19.9) | 0.419 |
| Metabolic syndrome, No. (%) | 4,679 (100) | 1,709 (100) | 2,970 (100) | **–** |
| Hypertension, No. (%) | 3,928 (84.0) | 1,444 (84.5) | 2,484 (83.6) | 0.442 |
| Hypertriglyceridemia, No. (%) | 1,916 (41.0) | 671 (39.3) | 1,245 (41.9) | 0.075 |
| Low HDL, No. (%) | 1,892 (40.4) | 777 (45.5) | 1,115 (37.5) | **0.001** |
| Hyperglycemia, No. (%) | 3,096 (66.2) | 1,105 (64.7) | 1,991 (67.0) | 0.098 |
| Central obesity, No. (%) | 3,768 (80.5) | 1,341 (78.5) | 2,427 (81.7) | **0.007** |
| Medication use, No. (%) |  |  |  |  |
| Insulin or other antidiabetic drugs | 1,138 (24.3) | 327 (19.1) | 811 (27.3) | **0.001** |
| Antihypertensive agents | 3,702 (79.1) | 1,381 (80.8) | 2,321 (78.1) | **0.031** |
| Statins or other hypolipidemic drugs | 2,404 (51.4) | 844 (49.4) | 1,560 (52.5) | **0.039** |
| **Lifestyle variables** |  |  |  |  |
| Physical activity, mean (SD), METs/min/d | 360.3 ± 330.2 | 397.0 ± 333.2 | 339.2 ± 326.7 | **0.001** |
| Sedentary time, mean (SD), h/d | 6.1 ± 1.9 | 5.7 ± 1.9 | 6.3 ± 1.9 | **0.001** |
| Smoking status, No. (%) |  |  |  |  |
| Current smoker | 609 (13.0) | 222 (13.0) | 387 (13.0) | 0.809 |
| Former smoker | 2,015 (43.1) | 750 (43.9) | 1,265 (42.6) |  |
| Never smoker | 2,055 (43.9) | 737 (43.1) | 1,318 (44.4) |  |
| **Dietary variables** |  |  |  |  |
| Alcohol intake, mean (SD), g/d | 11.6 ± 15.7 | 12.3 ± 16.1 | 11.2 ± 15.5 | **0.020** |
| Energy intake, mean (SD), kcal/d | 2,429 ± 621 | 2,436 ± 578 | 2,425 ± 645 | 0.569 |
| Abbreviations: BMI, body mass index. DXA, dual energy x-ray absorptiometry. HDL, high-density lipoprotein cholesterol. METs, metabolic equivalents.  ^a^Data are presented as n (%) or mean ± SD for categorical and continuous variables, respectively.  ^b^Participants selected for DXA measurements  ^c^Participants non-selected for DXA measurements  ^d^Only those participants with available data in all variables at baseline were included. Initially, 6,874 participants formed the overall population, with 2,520 being selected for DXA measurements and 4,354 non selected. | | | | |

| **Table S4.- Baseline sarcopenic obesity and cognitive performance over two, four, and six years of follow-up after excluding lost to follow-up participants (complete case analysis)^a^** | | | | | | | | | |
| --- | --- | --- | --- | --- | --- | --- | --- | --- | --- |
|  |  | **Basic model** | | | | **Multivariable-adjusted model** | | | |
| **Variable** | | **Changes in non-sarcopenic obesity participants** | **Changes in sarcopenic obesity participants** | **Mean difference in changes** | **P-value^c^** | **Changes in non-sarcopenic obesity participants** | **Changes in sarcopenic obesity participants** | **Mean difference in changes** | **P-value^c^** |
| **Global cognitive function** | | |  |  |  |  |  |  |  |
| Year 2 vs baseline | Mean [95% CI]^b^ | 0.7 [–0.1 to 1.5] | **1.9 [0.8 to 3.0]** | 1.3 [–0.1 to 2.6] | **0.025** | 0.7 [–0.1 to 1.4] | **1.9 [0.7 to 3.0]** | 1.2 [–0.1 to 2.6] | **0.030** |
| Year 4 vs baseline | Mean [95% CI]^b^ | –0.4 [–1.2 to 0.4] | 0.7 [–0.4 to 1.8] | 1.1 [–0.2 to 2.5] |  | –0.5 [–1.3 to 0.3] | 0.6 [–0.5 to 1.7] | 1.1 [–0.3 to 2.4] |  |
| Year 6 vs baseline | Mean [95% CI]^b^ | **–2.0 [–2.8 to –1.3]** | **–2.6 [–3.7 to –1.4]** | –0.5 [–1.9 to 0.8] |  | **–2.1 [–3.0 to –1.3]** | **–2.7 [–3.8 to –1.6]** | –0.5 [–1.9 to 0.8] |  |
| **General cognitive function** | | |  |  |  |  |  | |  |
| Year 2 vs baseline | Mean [95% CI]^b^ | **1.4 [0.1 to 2.7]** | 1.8 [–0.1 to 3.6] | 0.3 [–1.9 to 2.6] | **0.041** | **1.4 [0.1 to 2.7]** | 1.8 [–0.1 to 3.6] | 0.3 [–1.9 to 2.6] | **0.040** |
| Year 4 vs baseline | Mean [95% CI]^b^ | 0.3 [–1.0 to 1.6] | 1.7 [–0.1 to 3.6] | 1.4 [–0.8 to 3.7] |  | 0.3 [–1.0 to 1.6] | 1.7 [–0.1 to 3.6] | 1.4 [–0.8 to 3.7] |  |
| Year 6 vs baseline | Mean [95% CI]^b^ | **–3.6 [–4.9 to –2.3]** | **–5.4 [–7.3 to –3.6]** | –1.8 [–4.0 to 0.5] |  | **–3.6 [–4.9 to –2.3]** | **–5.4 [–7.3 to –3.6]** | –1.8 [–4.0 to 0.5] |  |
| **Executive function** | | | | | | | | | |
| Year 2 vs baseline | Mean [95% CI]^b^ | **1.3 [0.5 to 2.0]** | **2.9 [1.7 to 4.0]** | **1.6 [0.2 to 2.9]** | **0.044** | **1.2 [0.4 to 2.0]** | **2.7 [1.6 to 3.8]** | **1.5 [0.2 to 2.9]** | 0.055 |
| Year 4 vs baseline | Mean [95% CI]^b^ | 0.3 [–0.5 to 1.1] | 1.0 [–0.2 to 2.0] | 0.6 [–0.8 to 1.9] |  | 0.2 [–0.7 to 1.0] | 0.7 [–0.4 to 1.8] | 0.5 [–0.8 to 1.9] |  |
| Year 6 vs baseline | Mean [95% CI]^b^ | –0.1 [–0.9 to 0.7] | –0.3 [–1.4 to –0.8] | –0.2 [–1.6 to 1.1] |  | –0.3 [–1.1 to 0.5] | –0.5 [–1.6 to 0.6] | –0.2 [–1.6 to 1.1] |  |
| **Attention** | |  |  |  |  |  |  |  |  |
| Year 2 vs baseline | Mean [95% CI]^b^ | **–1.6 [–2.8 to –0.5]** | –0.9 [–2.6 to 0.7] | 0.7 [–1.3 to 2.7] | 0.768 | **–1.6 [–2.8 to –0.4]** | –0.8 [–2.4 to 0.8] | 0.8 [–1.2 to 2.8] | 0.765 |
| Year 4 vs baseline | Mean [95% CI]^b^ | **–2.2 [–3.3 to –1.0]** | –1.1 [–2.7 to 0.6] | 1.1 [–1.0 to 3.1] |  | **–2.2 [–3.4 to –1.1]** | –1.0 [–2.6 to 0.7] | 1.3 [–0.7 to 3.3] |  |
| Year 6 vs baseline | Mean [95% CI]^b^ | **–2.8 [–3.9 to –1.6]** | **–2.2 [–3.8 to –0.5]** | 0.5 [–1.5 to 2.6] |  | **–2.8 [–4.1 to –1.6]** | **–1.9 [–3.5 to –0.2]** | 1.0 [–1.0 to 3.0] |  |
| **Language** |  |  |  |  |  |  |  |  |  |
| Year 2 vs baseline | Mean [95% CI]^b^ | **1.5 [0.6 to 2.5]** | **2.4 [1.1 to 3.8]** | 1.0 [–0.7 to 2.6] | 0.107 | **1.4 [0.5 to 2.4]** | **2.3 [1.0 to 3.7]** | 0.9 [–0.8 to 2.5] | 0.119 |
| Year 4 vs baseline | Mean [95% CI]^b^ | **1.4 [0.5 to 2.3]** | **1.9 [0.5 to 3.2]** | 0.4 [–1.2 to 2.1] |  | **1.3 [0.3 to 2.2]** | **1.7 [0.3 to 3.0]** | 0.4 [–1.2 to 2.0] |  |
| Year 6 vs baseline | Mean [95% CI]^b^ | **1.6 [0.7 to 2.5]** | 0.6 [–0.8 to 1.9] | –1.0 [–2.7 to 0.6] |  | **1.5 [0.5 to 2.4]** | 0.4 [–1.0 to 1.7] | –1.1 [–2.7 to 0.6] |  |
| Abbreviations: CI, confidence interval.  ^a^Two-level linear mixed models were fitted with random intercepts at cluster family (as couples from the same household were randomized together), and individual participants to assess relationships between the baseline presence of sarcopenic obesity (yes or no) (exposure) and cognitive function composite scores (outcome) measured repeatedly over time (at each follow-up visit and for the overall follow-up period). An interaction term between baseline sarcopenic obesity (yes or no) and time, age (years), sex (male or female), and recruiting center were included as fixed effects in the basic models. Intervention group (control or intervention), baseline education level (primary or less, secondary, or college), marital status (single, divorced or separated, married, or widower), smoking status (current, former, or never), depressive symptomatology (yes or no), type 2 diabetes prevalence (yes or no), hypertension prevalence (yes or no), hypercholesterolemia prevalence (yes or no), physical activity (metabolic equivalents in minutes per day), and time varying sedentary time (hours per day), alcohol consumption (grams per day), and total energy intake (kilocalories per day) were additionally included as fixed effects in the multivariable-adjusted models. Data are presented as mean standardized values [95% CI]. Lost to follow-up participants with no information for cognitive performance composite scores were excluded. Significant differences (*p*<0.05) were highlighted in bold type. Overall (n=340); Non sarcopenic obesity (n=228); Sarcopenic obesity (n=112).  ^b^Expressed as multiples of 10^-1^ (x10^-1^).  ^c^P-values for the overall follow-up period. | | | | | | | | | |

| **Table S5. Baseline sarcopenic obesity compared with sarcopenia, or obesity, or overweight alone, and cognitive performance over two, four, and six years of follow-up^a^** | | | | | | | |
| --- | --- | --- | --- | --- | --- | --- | --- |
| **Variable** | | **Multivariable-adjusted model** | | | | | |
|  |  | **Sarcopenic obesity vs sarcopenia-only** | **P-value^c^** | **Sarcopenic obesity vs obesity-only** | **P-value^c^** | **Sarcopenic obesity vs overweight-only** | **P-value^c^** |
| **Global cognitive function** | | |  |  |  |  |  |
| Year 2 vs baseline | Mean [95% CI]^b^ | 1.3 [–0.1 to 2.8] | 0.172 | 0.5 [–0.4 to 1.4] | 0.071 | 0.4 [–0.6 to 1.4] | 0.420 |
| Year 4 vs baseline | Mean [95% CI]^b^ | 0.8 [–0.9 to 2.4] |  | 0.3 [–0.7 to 1.3] |  | 0.3 [–0.8 to 1.4] |  |
| Year 6 vs baseline | Mean [95% CI]^b^ | –0.7 [–2.8 to 1.5] |  | –1.3 [–2.6 to 0.1] |  | –0.8 [–2.3 to 0.7] |  |
| **General cognitive function** | | |  |  |  |  |  |
| Year 2 vs baseline | Mean [95% CI]^b^ | 1.3 [–1.0 to 3.7] | 0.322 | –0.2 [–1.7 to 1.3] | **0.017** | 0.4 [–1.1 to 2.0] | 0.312 |
| Year 4 vs baseline | Mean [95% CI]^b^ | 1.0 [–1.6 to 3.7] |  | 0.7 [–0.8 to 2.3] |  | 0.1 [–1.6 to 1.7] |  |
| Year 6 vs baseline | Mean [95% CI]^b^ | –1.5 [–4.9 to 1.9] |  | **–3.0 [–5.2 to –0.8]** |  | –1.8 [–4.0 to 0.5] |  |
| **Executive function** | | | | | | | |
| Year 2 vs baseline | Mean [95% CI]^b^ | 1.3 [–0.2 to 2.9] | 0.202 | **0.9 [0.1 to 1.9]** | 0.147 | 0.1 [–1.0 to 1.0] | 0.918 |
| Year 4 vs baseline | Mean [95% CI]^b^ | 0.7 [–1.0 to 2.4] |  | 0.2 [–0.8 to 1.2] |  | 0.2 [–1.0 to 1.3] |  |
| Year 6 vs baseline | Mean [95% CI]^b^ | –0.3 [–2.1 to 1.5] |  | 0.3 [–0.7 to 1.4] |  | –0.3 [–1.5 to 0.9] |  |
| **Attention** | |  |  |  |  |  |  |
| Year 2 vs baseline | Mean [95% CI]^b^ | 0.3 [–1.8 to 2.5] | 0.805 | 0.1 [–1.3 to 1.3] | 0.655 | 0.8 [–0.7 to 2.3] | 0.640 |
| Year 4 vs baseline | Mean [95% CI]^b^ | 0.7 [–1.6 to 3.1] |  | 0.1 [–1.3 to 1.6] |  | 0.9 [–0.8 to 2.5] |  |
| Year 6 vs baseline | Mean [95% CI]^b^ | –0.5 [–3.0 to 2.0] |  | –0.8 [–2.4 to 0.7] |  | 0.2 [–1.6 to 2.0] |  |
| **Language** |  |  |  |  |  |  |  |
| Year 2 vs baseline | Mean [95% CI]^b^ | **1.0 [–0.9 to 2.9]** | 0.377 | 0.7 [–0.3 to 1.8] | 0.123 | 0.1 [–1.1 to 1.3] | 0.537 |
| Year 4 vs baseline | Mean [95% CI]^b^ | **–0.7 [–2.8 to 1.3]** |  | –0.3 [–1.5 to 0.9] |  | 0.4 [–0.9 to 1.8] |  |
| Year 6 vs baseline | Mean [95% CI]^b^ | –0.4 [–2.6 to 1.8] |  | –0.7 [–2.0 to 0.6] |  | –0.7 [–2.2 to 0.8] |  |
| Abbreviations: CI, confidence interval.  ^a^Two-level linear mixed models were fitted with random intercepts at cluster family (as couples from the same household were randomized together), and individual participants to assess relationships between the baseline presence of sarcopenic obesity, compared with sarcopenia, or obesity, or overweight alone (exposure) and cognitive function composite scores (outcome) measured repeatedly over time (at each follow-up visit and for the overall follow-up period). An interaction term between baseline exposure and time, age (years), sex (male or female), recruiting center, intervention group (control or intervention), baseline education level (primary or less, secondary, or college), marital status (single, divorced or separated, married, or widower), smoking status (current, former, or never), depressive symptomatology (yes or no), type 2 diabetes prevalence (yes or no), hypertension prevalence (yes or no), hypercholesterolemia prevalence (yes or no), physical activity (metabolic equivalents in minutes per day), and time varying sedentary time (hours per day), alcohol consumption (grams per day), and total energy intake (kilocalories per day) were included as fixed effects in the multivariable-adjusted models. Data are presented as intergroup mean differences standardized values [95% CI]. Significant differences (*p*<0.05) were highlighted in bold type. Overall (n=1,097); Sarcopenic obesity (n=364); Sarcopenia-only (n=75); Obesity-only (n=398); Overweight-only (n=260).  ^b^Expressed as multiples of 10^-1^ (x10^-1^).  ^c^P-values for the overall follow-up period. | | | | | | | |

| **Table S6. Baseline sarcopenia or obesity alone, compared with participants without these conditions, and cognitive performance over two, four, and six years of follow-up^a^** | | | | | |
| --- | --- | --- | --- | --- | --- |
| **Variable** | | **Multivariable-adjusted model** | | | |
|  |  | **Sarcopenia-only vs non-sarcopenia^c^** | **P-value^e^** | **Obesity-only vs non-obesity^d^** | **P-value^e^** |
| **Global cognitive function** | | |  |  |  |
| Year 2 vs baseline | Mean [95% CI]^b^ | –0.9 [2.5 to 0.6] | 0.696 | 0.2 [–0.8 to 1.1] | 0.902 |
| Year 4 vs baseline | Mean [95% CI]^b^ | –0.5 [–2.2 to 1.2] |  | 0.2 [–0.9 to 1.2] |  |
| Year 6 vs baseline | Mean [95% CI]^b^ | –0.5 [–2.7 to 1.8] |  | 0.6 [–0.9 to 2.0] |  |
| **General cognitive function** | | |  |  |  |
| Year 2 vs baseline | Mean [95% CI]^b^ | –1.5 [–4.0 to 1.1] | 0.733 | 1.0 [–0.5 to 2.6] | 0.291 |
| Year 4 vs baseline | Mean [95% CI]^b^ | –0.7 [–3.5 to 2.1]- |  | –0.3 [–2.0 to 1.4] |  |
| Year 6 vs baseline | Mean [95% CI]^b^ | –0.9 [–4.5 to 2.7] |  | 1.3 [–1.0 to 3.7] |  |
| **Executive function** | | | | | |
| Year 2 vs baseline | Mean [95% CI]^b^ | –0.8 [–2.3 to 0.7] | 0.497 | –0.7 [–1.6 to 0.2] | 0.287 |
| Year 4 vs baseline | Mean [95% CI]^b^ | –0.6 [–2.2 to 1.1] |  | 0.1 [–1.0 to 1.1] |  |
| Year 6 vs baseline | Mean [95% CI]^b^ | 0.4 [–1.3 to 2.2] |  | –0.7 [–1.8 to 0.4] |  |
| **Attention** | |  |  |  |  |
| Year 2 vs baseline | Mean [95% CI]^b^ | –0.1 [–2.4 to 2.2] | 0.995 | 0.8 [–0.6 to 2.2] | 0.607 |
| Year 4 vs baseline | Mean [95% CI]^b^ | –0.2 [–2.7 to 2.3] |  | 0.8 [–0.7 to 2.4] |  |
| Year 6 vs baseline | Mean [95% CI]^b^ | 0.2 [–2.5 to 2.9] |  | 0.9 [–0.8 to 2.6] |  |
| **Language** |  |  |  |  |  |
| Year 2 vs baseline | Mean [95% CI]^b^ | –0.5 [–2.3 to 1.3] | 0.749 | –0.5 [–1.6 to 0.6] | 0.486 |
| Year 4 vs baseline | Mean [95% CI]^b^ | 0.6 [–1.4 to 2.6] |  | 0.5 [–0.7 to 1.7] |  |
| Year 6 vs baseline | Mean [95% CI]^b^ | –0.3 [–2.4 to 1.9] |  | 0.1 [–1.3 to 1.4] |  |
| Abbreviations: CI, confidence interval.  ^a^Two-level linear mixed models were fitted with random intercepts at cluster family (as couples from the same household were randomized together), and individual participants to assess relationships between the baseline presence of sarcopenia or obesity alone, compared with participants without these conditions (exposure) and cognitive function composite scores (outcome) measured repeatedly over time (at each follow-up visit and for the overall follow-up period). An interaction term between baseline exposure and time, age (years), sex (male or female), recruiting center, intervention group (control or intervention), baseline education level (primary or less, secondary, or college), marital status (single, divorced or separated, married, or widower), smoking status (current, former, or never), depressive symptomatology (yes or no), type 2 diabetes prevalence (yes or no), hypertension prevalence (yes or no), hypercholesterolemia prevalence (yes or no), physical activity (metabolic equivalents in minutes per day), and time varying sedentary time (hours per day), alcohol consumption (grams per day), and total energy intake (kilocalories per day) were included as fixed effects in the multivariable-adjusted models. Data are presented as intergroup mean differences standardized values [95% CI]. Significant differences (*p*<0.05) were highlighted in bold type.  ^b^Expressed as multiples of 10^-1^ (x10^-1^).  ^e^Non-sarcopenia is defined as obesity and overweight. Overall (n=733); Sarcopenia-only (n=75); Non-sarcopenia (n=658).  ^d^Non-obesity is defined as overweight and sarcopenia. Overall (n=733); Obesity-only (n=398); Non-obesity (n=335).  ^e^P-values for the overall follow-up period. | | | | | |

| **Table S7. Baseline sarcopenic obesity and cognitive performance over two, four, and six years of follow-up by age^a^** | | | | | | | | | |
| --- | --- | --- | --- | --- | --- | --- | --- | --- | --- |
| **Variable** | | **Multivariable-adjusted model** | | | | | | | |
|  |  | **< median age^b^** | | | | **≥ Median age^c^** | | | |
|  |  | **Sarcopenic obesity vs non-sarcopenic obesity^b^** | | | **P-value^e^** | **Sarcopenic obesity vs non-sarcopenic obesity^c^** | | | **P-value^e^** |
| **Global cognitive function** | | |  |  |  |  |  |  |  |
| Year 2 vs baseline | Mean [95% CI]^d^ | 0.3 [–0.7 to 1.4] | | | 0.632 | 0.9 [–0.3 to 2.0] | | | 0.078 |
| Year 4 vs baseline | Mean [95% CI]^d^ | 0.1 [–1.2 to 1.2] | | |  | 0.8 [–0.5 to 2.1] | | |  |
| Year 6 vs baseline | Mean [95% CI]^d^ | –0.8 [–2.4 to 0.9] | | |  | –1.2 [–2.9 to 0.6] | | |  |
| **General cognitive function** | | |  |  |  |  |  | |  |
| Year 2 vs baseline | Mean [95% CI]^d^ | 0.1 [–1.8 to 1.8] | | | 0.222 | 0.3 [–1.5 to 2.2] | | | 0.178 |
| Year 4 vs baseline | Mean [95% CI]^d^ | 0.5 [–1.5 to 2.5] | | |  | 0.6 [–1.4 to 2.6] | | |  |
| Year 6 vs baseline | Mean [95% CI]^d^ | –2.4 [–5.1 to 0.3] | | |  | –2.4 [–5.1 to 0.3] | | |  |
| **Executive function** | | | | | | | | | |
| Year 2 vs baseline | Mean [95% CI]^d^ | 0.3 [–0.8 to 1.4] | | | 0.931 | 1.1 [0.1 to 2.2] | | | 0.157 |
| Year 4 vs baseline | Mean [95% CI]^d^ | 0.1 [–1.2 to 1.4] | | |  | 0.5 [–0.7 to 1.7] | | |  |
| Year 6 vs baseline | Mean [95% CI]^d^ | 0.4 [–1.0 to 1.8] | | |  | –0.1 [–1.4 to 1.2] | | |  |
| **Attention** | |  |  |  |  |  |  |  |  |
| Year 2 vs baseline | Mean [95% CI]^d^ | 0.4 [–1.0 to 1.7] | | | 0.957 | 0.5 [–1.4 to 2.3] | | | 0.494 |
| Year 4 vs baseline | Mean [95% CI]^d^ | 0.1 [–1.5 to 1.6] | | |  | 1.0 [–1.0 to 3.1] | | |  |
| Year 6 vs baseline | Mean [95% CI]^d^ | 0.1 [–1.6 to 1.8] | | |  | –0.8 [–3.0 to 1.5] | | |  |
| **Language** |  |  |  |  |  |  |  |  |  |
| Year 2 vs baseline | Mean [95% CI]^d^ | 0.7 [–0.7 to 2.0] | | | 0.384 | 0.4 [–0.9 to 1.8] | | | 0.739 |
| Year 4 vs baseline | Mean [95% CI]^d^ | 0.1 [–1.4 to 1.6] | | |  | –0.1 [–1.6 to 1.4] | | |  |
| Year 6 vs baseline | Mean [95% CI]^d^ | –0.8 [–2.5 to 0.9] | | |  | –0.4 [–2.0 to 1.2] | | |  |
| Abbreviations: CI, confidence interval.  ^a^Two-level linear mixed models were fitted with random intercepts at cluster family (as couples from the same household were randomized together), and individual participants to assess relationships between the baseline presence of sarcopenic obesity (yes or no) (exposure) and cognitive function composite scores (outcome) measured repeatedly over time (at each follow-up visit and for the overall follow-up period). An interaction term between baseline sarcopenic obesity (yes or no) and time, sex (male or female), recruiting center, intervention group (control or intervention), baseline education level (primary or less, secondary, or college), marital status (single, divorced or separated, married, or widower), smoking status (current, former, or never), depressive symptomatology (yes or no), type 2 diabetes prevalence (yes or no), hypertension prevalence (yes or no), hypercholesterolemia prevalence (yes or no), physical activity (metabolic equivalents in minutes per day), and time varying sedentary time (hours per day), alcohol consumption (grams per day), and total energy intake (kilocalories per day) were additionally included as fixed effects in the multivariable-adjusted models. Data are presented as intergroup mean differences standardized values [95% CI]. Significant differences (*p*<0.05) were highlighted in bold type.  ^b^For participants < median age: Overall (n=545); Non-sarcopenic obesity (n=376); Sarcopenic obesity (n=169).  ^c^For participants ≥ median age: Overall (n=552); Non-sarcopenic obesity (n=357); Sarcopenic obesity (n=195).  ^d^Expressed as multiples of 10^-1^ (x10^-1^).  ^e^P-values for the overall follow-up period. | | | | | | | | | |

| **Table S8. Baseline sarcopenic obesity and cognitive performance over two, four, and six years of follow-up by sex^a^** | | | | | | | | | |
| --- | --- | --- | --- | --- | --- | --- | --- | --- | --- |
| **Variable** | | **Multivariable-adjusted model** | | | | | | | |
|  |  | **Male^b^** | | | | **Female^c^** | | | |
|  |  | **Sarcopenic obesity vs non-sarcopenic obesity^b^** | | | **P-value^e^** | **Sarcopenic obesity vs non-sarcopenic obesity^c^** | | | **P-value^e^** |
| **Global cognitive function** | | |  |  |  |  |  |  |  |
| Year 2 vs baseline | Mean [95% CI]^d^ | 0.9 [–0.1 to 1.9] | | | **0.047** | 0.3 [–0.9 to 1.5] | | | 0.486 |
| Year 4 vs baseline | Mean [95% CI]^d^ | 0.3 [–0.8 to 1.5] | | |  | 0.6 [–0.7 to 2.0] | | |  |
| Year 6 vs baseline | Mean [95% CI]^d^ | –1.2 [–2.7 to 0.4] | | |  | –0.8 [–2.7 to 1.0] | | |  |
| **General cognitive function** | | |  |  |  |  |  | |  |
| Year 2 vs baseline | Mean [95% CI]^d^ | –0.2 [–1.9 to 1.5] | | | **0.005** | 0.7 [–1.3 to 2.6] | | | 0.720 |
| Year 4 vs baseline | Mean [95% CI]^d^ | 0.3 [–1.6 to 2.2] | | |  | 1.0 [–1.1 to 3.1] | | |  |
| Year 6 vs baseline | Mean [95% CI]^d^ | **–4.2 [–6.8 to –1.7]** | | |  | –0.4 [–3.2 to 2.5] | | |  |
| **Executive function** | | | | | | | | | |
| Year 2 vs baseline | Mean [95% CI]^d^ | **1.4 [0.3 to 2.5]** | | | 0.072 | 0.1 [–1.1 to 1.2] | | | 0.545 |
| Year 4 vs baseline | Mean [95% CI]^d^ | 0.3 [–0.9 to 1.6] | | |  | 0.4 [–0.8 to 1.7] | | |  |
| Year 6 vs baseline | Mean [95% CI]^d^ | 0.8 [–0.5 to 2.1] | | |  | –0.7 [–2.0 to 0.7] | | |  |
| **Attention** | |  |  |  |  |  |  |  |  |
| Year 2 vs baseline | Mean [95% CI]^d^ | 0.5 [–0.9 to 1.9] | | | 0.572 | 0.3 [–1.6 to 2.3] | | | 0.693 |
| Year 4 vs baseline | Mean [95% CI]^d^ | 1.1 [–0.5 to 2.7] | | |  | 0.1 [–2.0 to 2.2] | | |  |
| Year 6 vs baseline | Mean [95% CI]^d^ | 0.2 [–1.6 to 1.9] | | |  | –1.0 [–3.3 to 1.3] | | |  |
| **Language** |  |  |  |  |  |  |  |  |  |
| Year 2 vs baseline | Mean [95% CI]^d^ | 0.9 [–0.4 to 2.3] | | | 0.346 | 0.1 [–1.2 to 1.5] | | | 0.667 |
| Year 4 vs baseline | Mean [95% CI]^d^ | 0.1 [–1.3 to 1.6] | | |  | –0.1 [–1.6 to 1.4] | | |  |
| Year 6 vs baseline | Mean [95% CI]^d^ | –0.4 [–2.0 to 1.2] | | |  | –0.9 [–2.5 to 0.8] | | |  |
| Abbreviations: CI, confidence interval.  ^a^Two-level linear mixed models were fitted with random intercepts at cluster family (as couples from the same household were randomized together), and individual participants to assess relationships between the baseline presence of sarcopenic obesity (yes or no) (exposure) and cognitive function composite scores (outcome) measured repeatedly over time (at each follow-up visit and for the overall follow-up period). An interaction term between baseline sarcopenic obesity (yes or no) and time, age (years), recruiting center, intervention group (control or intervention), baseline education level (primary or less, secondary, or college), marital status (single, divorced or separated, married, or widower), smoking status (current, former, or never), depressive symptomatology (yes or no), type 2 diabetes prevalence (yes or no), hypertension prevalence (yes or no), hypercholesterolemia prevalence (yes or no), physical activity (metabolic equivalents in minutes per day), and time varying sedentary time (hours per day), alcohol consumption (grams per day), and total energy intake (kilocalories per day) were additionally included as fixed effects in the multivariable-adjusted models. Data are presented as intergroup mean differences standardized values [95% CI]. Significant differences (*p*<0.05) were highlighted in bold type.  ^b^For male participants: Overall (n=591); Non-sarcopenic obesity (n=410); Sarcopenic obesity (n=181).  ^c^For female participants: Overall (n=506); Non-sarcopenic obesity (n=323); Sarcopenic obesity (n=183).  ^d^Expressed as multiples of 10^-1^ (x10^-1^).  ^e^P-values for the overall follow-up period. | | | | | | | | | |

| **Table S9. Baseline sarcopenic obesity and subtle cognitive impairment over two, four, and six years after excluding lost to follow-up participants (complete case analysis)^a^** | | | | |
| --- | --- | --- | --- | --- |
| **Variable** | **Basic model** | | **Multivariable-adjusted model** | |
|  | **Sarcopenic obesity** | **Overall P-value^b^** | **Sarcopenic obesity** | **Overall P-value^b^** |
| **Global cognitive function** |  |  |  |  |
| Year 2 vs baseline | **0.2 [0.1 to 0.6]** | **0.022** | **0.2 [0.1 to 0.7]** | **0.019** |
| Year 4 vs baseline | 0.7 [0.2 to 2.2] |  | 0.7 [0.2 to 2.2] |  |
| Year 6 vs baseline | 1.0 [0.3 to 3.3] |  | 1.2 [0.4 to 3.6] |  |
| **General cognitive function** |  |  |  |  |
| Year 2 vs baseline | 0.9 [0.4 to 2.1] | 0.843 | 0.9 [0.4 to 2.1] | 0.843 |
| Year 4 vs baseline | 0.8 [0.4 to 1.9] |  | 0.8 [0.4 to 1.9] |  |
| Year 6 vs baseline | 1.2 [0.5 to 2.7] |  | 1.2 [0.5 to 2.7] |  |
| **Executive function** |  |  |  |  |
| Year 2 vs baseline | 0.2 [0.1 to 0.8] | 0.059 | 0.2 [0.1 to 0.8] | 0.070 |
| Year 4 vs baseline | 0.7 [0.2 to 2.1] |  | 0.7 [0.2 to 2.2] |  |
| Year 6 vs baseline | 1.1 [0.3 to 3.2] |  | 1.1 [0.4 to 3.6] |  |
| **Attention** |  |  |  |  |
| Year 2 vs baseline | 0.3 [0.1 to 0.9] | 0.072 | 0.5 [0.2 to 1.3] | 0.079 |
| Year 4 vs baseline | 0.3 [0.1 to 0.8] |  | 0.5 [0.2 to 1.3] |  |
| Year 6 vs baseline | 0.5 [0.2 to 1.3] |  | 0.5 [0.2 to 1.3] |  |
| **Language** |  |  |  |  |
| Year 2 vs baseline | 0.9 [0.3 to 2.6] | 0.594 | 0.8 [0.3 to 2.4] | 0.600 |
| Year 4 vs baseline | 0.5 [0.2 to 1.4] |  | 0.5 [0.2 to 1.4] |  |
| Year 6 vs baseline | 0.7 [0.3 to 2.0] |  | 0.7 [0.3 to 2.1] |  |
| Abbreviations: CI, confidence interval. OR, odds ratio.  ^a^Two-level logistic mixed models were fitted with random intercepts at cluster family (as couples from the same household were randomized together), and individual participants to assess relationships between the baseline presence of sarcopenic obesity (yes or no) (exposure) and subtle cognitive impairment (outcome) measured repeatedly over time (at each follow-up visit and for the overall follow-up period). An interaction term between baseline sarcopenic obesity (yes or no) and time, age (years), sex (male or female), and recruiting center were included as fixed effects in the basic models. Intervention group (control or intervention), baseline education level (primary or less, secondary, or college), marital status (single, divorced or separated, married, or widower), smoking status (current, former, or never), depressive symptomatology (yes or no), type 2 diabetes prevalence (yes or no), hypertension prevalence (yes or no), hypercholesterolemia prevalence (yes or no), physical activity (metabolic equivalents in minutes per day), and time varying sedentary time (hours per day), alcohol consumption (grams per day), and total energy intake (kilocalories per day) were additionally included as fixed effects in the multivariable-adjusted models. The non presence of sarcopenic obesity was considered as 1 (reference). Subtle cognitive impairment was defined as presenting a cognitive performance composite score less than or equal to the baseline 0.5 SD of the distribution for each z-score. Data are presented as OR standardized values [95% CI]. Lost to follow-up participants with no information for cognitive performance composite scores were excluded. Significant values (*p*<0.05) were highlighted in bold type. Overall (n=340); Non sarcopenic obesity (n=228); Sarcopenic obesity (n=112).  ^b^P-value represents the intervention group effects assessed for the overall follow-up period. | | | | |

| **Table S10. Baseline sarcopenic obesity compared with sarcopenia, or obesity, or overweight alone, and subtle cognitive impairment over two, four, and six years of follow-up participants^a^** | | | | | | |
| --- | --- | --- | --- | --- | --- | --- |
| **Variable** | **Multivariable-adjusted model** | | | | | |
|  | **Sarcopenic obesity vs sarcopenia-only** | **Overall P-value^b^** | **Sarcopenic obesity vs obesity-only** | **Overall P-value^b^** | **Sarcopenic obesity vs overweight-only** | **Overall P-value^b^** |
| **Global cognitive function** | |  |  |  |  |  |
| Year 2 vs baseline | 0.2 [0.1 to 1.2] | 0.202 | 0.6 [0.3 to 1.5] | 0.090 | 0.6 [0.3 to 1.5] | 0.312 |
| Year 4 vs baseline | 0.9 [1.7 to 5.1] |  | 0.5 [0.2 to 1.2] |  | 0.5 [0.2 to 1.2] |  |
| Year 6 vs baseline | 1.4 [0.2 to 11.1] |  | 2.2 [0.7 to 7.0] |  | 1.2 [0.7 to 4.1] |  |
| **General cognitive function** | |  |  |  |  |  |
| Year 2 vs baseline | 0.7 [0.2 to 2.0] | 0.834 | 0.7 [0.4 to 1.2] | 0.189 | 0.8 [0.4 to 1.4] | 0.761 |
| Year 4 vs baseline | 0.8 [0.3 to 2.4] |  | 0.7 [0.4 to 1.3] |  | 1.0 [0.5 to 2.0] |  |
| Year 6 vs baseline | 1.2 [0.3 to 4.4] |  | 1.4 [0.7 to 3.0] |  | 1.1 [0.5 to 2.7] |  |
| **Executive function** | |  |  |  |  |  |
| Year 2 vs baseline | 0.5 [0.1 to 1.8] | 0.284 | 0.7 [0.3 to 1.4] | 0.769 | 0.6 [0.2 to 1.3] | 0.488 |
| Year 4 vs baseline | 1.4 [0.3 to 6.5] |  | 1.0 [0.4 to 2.2] |  | 0.6 [0.2 to 1.5] |  |
| Year 6 vs baseline | 1.9 [0.4 to 8.8] |  | 0.8 [0.4 to 2.0] |  | 1.0 [0.4 to 2.7] |  |
| **Attention** |  |  |  |  |  |  |
| Year 2 vs baseline | 1.5 [0.4 to 5.4] | 0.345 | 1.1 [0.6 to 2.0] | 0.234 | 0.7 [0.4 to 1.5] | 0.741 |
| Year 4 vs baseline | 0.5 [0.1 to 1.7] |  | 0.9 [0.5 to 1.7] |  | 0.9 [0.4 to 2.1] |  |
| Year 6 vs baseline | 1.3 [0.3 to 5.4] |  | 1.8 [0.9 to 3.7] |  | 0.7 [0.3 to 1.6] |  |
| **Language** |  |  |  |  |  |  |
| Year 2 vs baseline | 1.9 [0.4 to 8.8] | 0.571 | 1.0 [0.5 to 1.9] | 0.987 | 0.7 [0.3 to 1.6] | 0.523 |
| Year 4 vs baseline | 0.6 [0.1 to 2.6] |  | 1.1 [0.5 to 2.3] |  | 0.5 [0.2 to 1.2] |  |
| Year 6 vs baseline | 1.1 [0.2 to 4.8] |  | 1.1 [0.5 to 2.5] |  | 0.7 [0.3 to 1.7] |  |
| Abbreviations: CI, confidence interval. OR, odds ratio.  ^a^Two-level logistic mixed models were fitted with random intercepts at cluster family (as couples from the same household were randomized together), and individual participants to assess relationships between the baseline presence of sarcopenic obesity, compared with sarcopenia, or obesity, or overweight alone (exposure) and subtle cognitive impairment (outcome) measured repeatedly over time (at each follow-up visit and for the overall follow-up period). An interaction term between baseline exposure and time, age (years), sex (male or female), recruiting center, intervention group (control or intervention), baseline education level (primary or less, secondary, or college), marital status (single, divorced or separated, married, or widower), smoking status (current, former, or never), depressive symptomatology (yes or no), type 2 diabetes prevalence (yes or no), hypertension prevalence (yes or no), hypercholesterolemia prevalence (yes or no), physical activity (metabolic equivalents in minutes per day), and time varying sedentary time (hours per day), alcohol consumption (grams per day), and total energy intake (kilocalories per day) were included as fixed effects in the multivariable-adjusted models. Subtle cognitive impairment was defined as presenting a cognitive performance composite score less than or equal to the baseline 0.5 SD of the distribution for each z-score. Data are presented as OR standardized values [95% CI]. Significant values (*p*<0.05) were highlighted in bold type. Overall (n=1,097); Sarcopenic obesity (n=364); Sarcopenia-only (n=75); Obesity-only (n=398); Overweight-only (n=260).  ^b^P-value represents the intervention group effects assessed for the overall follow-up period. | | | | | | |

| **Table S11. Baseline sarcopenia or obesity compared with participants without these conditions and subtle cognitive impairment over two, four, and six years of follow-up^a^** | | | | |
| --- | --- | --- | --- | --- |
| **Variable** | **Multivariable-adjusted model** | | | |
|  | **Sarcopenia-only vs non-sarcopenia^b^** | **Overall P-value^d^** | **Obesity-only vs non-obesity^c^** | **Overall P-value^d^** |
| **Global cognitive function** |  |  |  |  |
| Year 2 vs baseline | 1.6 [0.4 to 6.2] | 0.415 | 0.7 [0.3 to 1.4] | 0.712 |
| Year 4 vs baseline | 0.5 [0.1 to 2.3] |  | 0.8 [0.3 to 1.7] |  |
| Year 6 vs baseline | 1.7 [0.3 to 9.4] |  | 0.6 [0.2 to 1.9] |  |
| **General cognitive function** |  |  |  |  |
| Year 2 vs baseline | 0.9 [0.3 to 2.6] | 0.967 | 1.0 [0.6 to 1.8] | 0.476 |
| Year 4 vs baseline | 0.8 [0.3 to 2.2] |  | 1.4 [0.8 to 2.5] |  |
| Year 6 vs baseline | 0.9 [0.2 to 3.0] |  | 0.8 [0.3 to 1.7] |  |
| **Executive function** |  |  |  |  |
| Year 2 vs baseline | 1.8 [0.5 to 6.3] | 0.532 | 1.1 [0.5 to 2.3] | 0.549 |
| Year 4 vs baseline | 0.7 [0.2 to 2.7] |  | 0.7 [0.3 to 1.5] |  |
| Year 6 vs baseline | 0.8 [0.2 to 3.4] |  | 1.2 [0.5 to 3.0] |  |
| **Attention** |  |  |  |  |
| Year 2 vs baseline | 1.2 [0.4 to 4.0] | 0.337 | 0.8 [0.4 to 1.6] | 0.375 |
| Year 4 vs baseline | 2.1 [0.6 to 7.4] |  | 0.9 [0.5 to 1.9] |  |
| Year 6 vs baseline | 2.9 [0.8 to 10.9] |  | 0.5 [0.2 to 1.1] |  |
| **Language** |  |  |  |  |
| Year 2 vs baseline | 0.6 [0.2 to 2.1] | 0.857 | 1.2 [0.6 to 2.5] | 0.369 |
| Year 4 vs baseline | 0.7 [0.2 to 2.9] |  | 0.6 [0.3 to 1.3] |  |
| Year 6 vs baseline | 0.9 [0.2 to 3.7] |  | 0.8 [0.3 to 1.8] |  |
| Abbreviations: CI, confidence interval. OR, odds ratio.  ^a^Two-level linear mixed models were fitted with random intercepts at cluster family (as couples from the same household were randomized together), and individual participants to assess relationships between the baseline presence of sarcopenia or obesity alone, compared with participants without these conditions (exposure) and subtle cognitive impairment (outcome) measured repeatedly over time (at each follow-up visit and for the overall follow-up period). An interaction term between baseline exposure and time, age (years), sex (male or female), recruiting center, intervention group (control or intervention), baseline education level (primary or less, secondary, or college), marital status (single, divorced or separated, married, or widower), smoking status (current, former, or never), depressive symptomatology (yes or no), type 2 diabetes prevalence (yes or no), hypertension prevalence (yes or no), hypercholesterolemia prevalence (yes or no), physical activity (metabolic equivalents in minutes per day), and time varying sedentary time (hours per day), alcohol consumption (grams per day), and total energy intake (kilocalories per day) were included as fixed effects in the multivariable-adjusted models. Subtle cognitive impairment was defined as presenting a cognitive performance composite score less than or equal to the baseline 0.5 SD of the distribution for each z-score. Data are presented as OR standardized values [95% CI]. Significant values (*p*<0.05) were highlighted in bold type.  ^b^Non-sarcopenia is defined as obesity and overweight. Overall (n=733); Sarcopenia-only (n=75); Non-sarcopenia (n=658).  ^c^Non-obesity is defined as overweight and sarcopenia. Overall (n=733); Obesity-only (n=398); Non-obesity (n=335).  ^d^P-values for the overall follow-up period. | | | | |

**
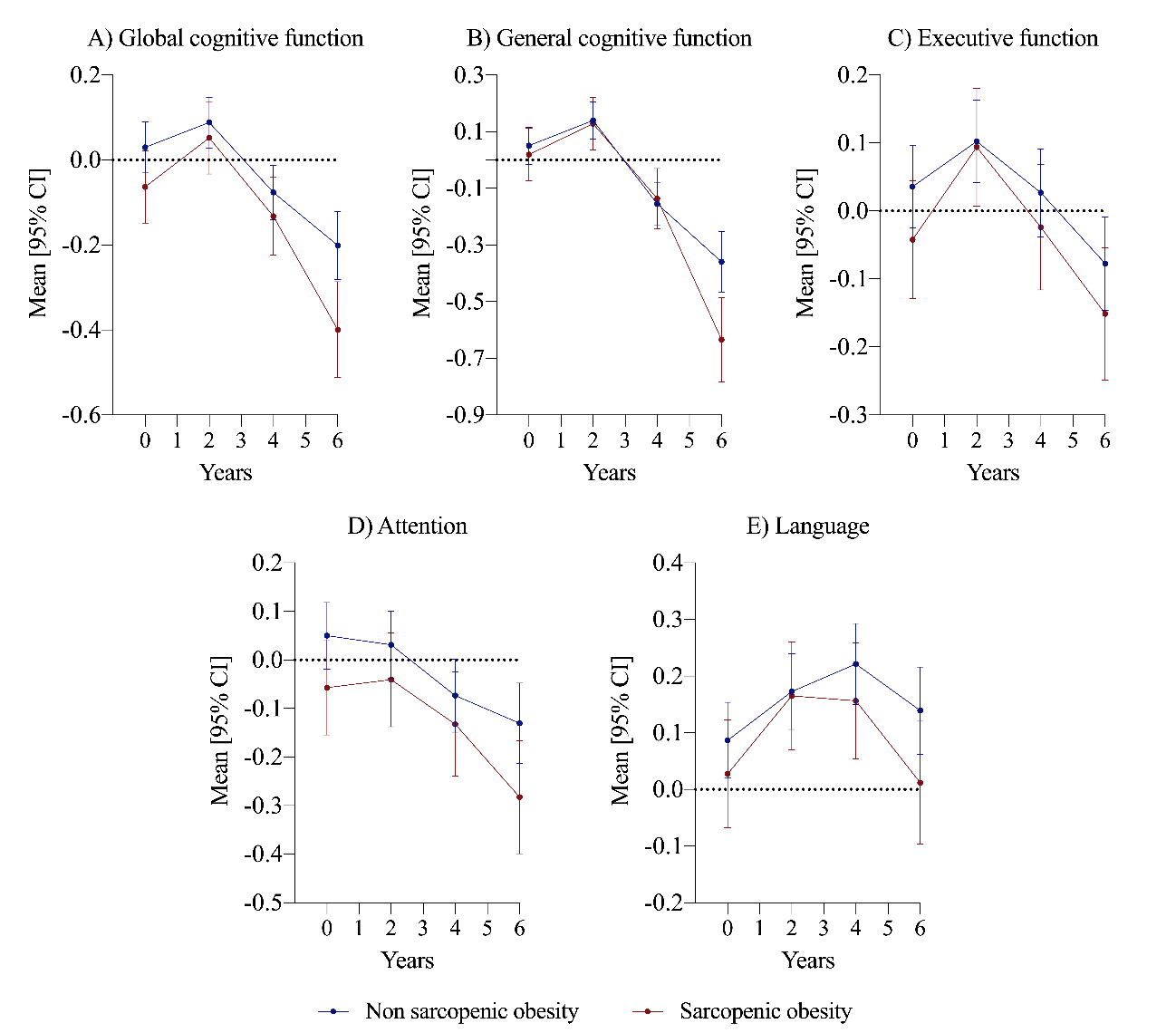
Fig. S1** **Adjusted means for baseline sarcopenic obesity and cognitive performance over two, four, and six years of follow-up.** Abbreviations: CI, confidence interval. Data are presented as means [95% CI]. Two-level linear mixed models were fitted with random intercepts at cluster family (as couples from the same household were randomized together), and individual participants to assess relationships between the baseline presence of sarcopenic obesity (yes or no) (exposure) and cognitive function composite scores (outcome) measured repeatedly over time (at each follow-up visit and for the overall follow-up period). An interaction term between baseline sarcopenic obesity (yes or no) and time, age (years), sex (male or female), and recruiting center were included as fixed effects in the basic models. Intervention group (control or intervention), baseline education level (primary or less, secondary, or college), marital status (single, divorced or separated, married, or widower), smoking status (current, former, or never), depressive symptomatology (yes or no), type 2 diabetes prevalence (yes or no), hypertension prevalence (yes or no), hypercholesterolemia prevalence (yes or no), physical activity (metabolic equivalents in minutes per day), and time varying sedentary time (hours per day), alcohol consumption (grams per day), and total energy intake (kilocalories per day) were additionally included as fixed effects in the multivariable-adjusted models.


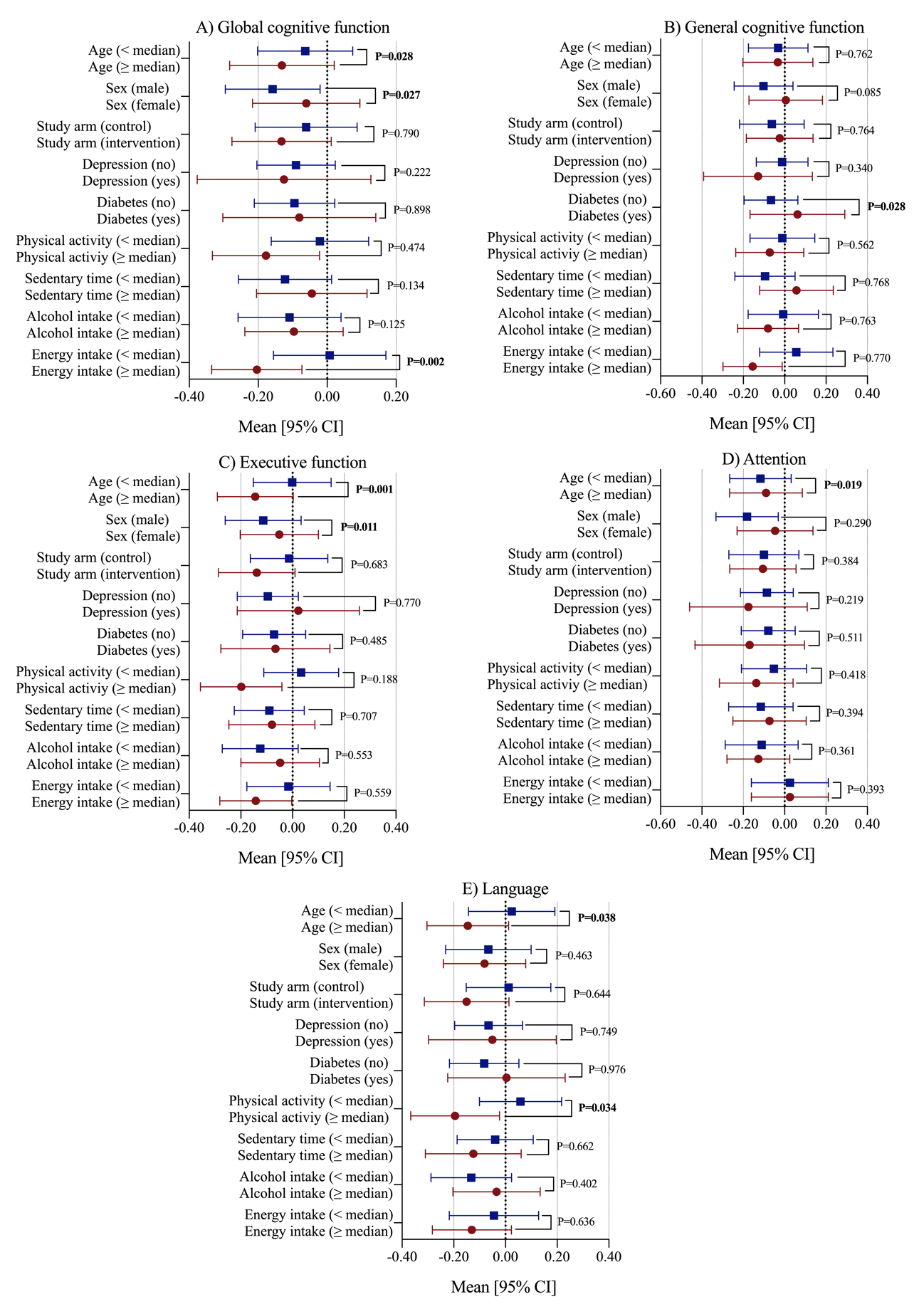


**Fig. S2** **A priory** **interaction for cognitive performance composite scores over six years by presence of baseline sarcopenic obesity with multiple baseline variables of the study.** Two-level linear mixed models were fitted with random intercepts at cluster family (as couples from the same household were randomized together), and individual participants to assess relationships between the baseline presence of sarcopenic obesity (yes or no) (exposure) and cognitive function composite scores (outcome) measured repeatedly over time (at each follow-up visit and for the overall follow-up period). An interaction term between baseline sarcopenic obesity (yes or no) and time, age (years), and sex (male or female), were included as fixed effects in the basic models. Intervention group (control or intervention), baseline education level (primary or less, secondary, or college), marital status (single, divorced or separated, married, or widower), smoking status (current, former, or never), depressive symptomatology (yes or no), type 2 diabetes prevalence (yes or no), hypertension prevalence (yes or no), hypercholesterolemia prevalence (yes or no), physical activity (metabolic equivalents in minutes per day), and time varying sedentary time (hours per day), alcohol consumption (grams per day), and total energy intake (kilocalories per day) were additionally included as fixed effects in the multivariable-adjusted models. Data are presented as mean standardized values [95% CI]. Significant values (*p*<0.05) were highlighted in bold type.
